# Supplementary material for: Multiancestry exome sequencing reveals INHBE mutations associated with favorable fat distribution and protection from diabetes
Source: Nat Commun. 2022 Aug 23;13:4844. doi: 10.1038/s41467-022-32398-7 (PMC9399235; doi:10.1038/s41467-022-32398-7)
Supplement: Supplementary file 1 — Supplementary Information [file 41467_2022_32398_MOESM1_ESM.pdf]

Supplementary Information for

Multiancestry exome sequencing reveals INHBE mutations associated with favorable fat distribution and protection from diabetes

Parsa Akbari[1,a], Olukayode A. Sosina[1,a], Jonas Bovijn[1,a], Karl Landheer[2], Jonas B. Nielsen[1], Minhee Kim[1], Senem Aykul[1], Tanima De[1], Mary E. Haas[1], George Hindy[1], Nan Lin[1], Ian R. Dinsmore[3], Jonathan Z. Luo[3], Stefanie Hectors[2], Benjamin Geraghty[1], Mary Germino[2], Lampros Panagis[2], Prodromos Parasoglou[2], Johnathon R. Walls[2], Gabor Halasz[2], Gurinder S. Atwal[2], Regeneron Genetics Center, DiscovEHR Collaboration, Marcus Jones[1], Michelle G. LeBlanc[1], Christopher D. Still[4], David J. Carey[4], Alice Giontella[5,6], Marju Orho-Melander[5], Jaime Berumen[7], Pablo Kuri-Morales[7,8], Jesus Alegre-Díaz[7], Jason M. Torres[9,10], Jonathan R. Emberson[9,10], Rory Collins[10], Daniel J. Rader[11], Brian Zambrowicz[2], Andrew J. Murphy[2], Suganthi Balasubramanian[1], John D. Overton[1], Jeffrey G. Reid[1], Alan R. Shuldiner[1], Michael Cantor[1], Goncalo R. Abecasis[1], Manuel A. R. Ferreira[1], Mark W. Sleeman[2], Viktoria Gusarova[2], Judith Altarejos[2], Charles Harris[2], Aris N. Economides[1,2], Vincent Idone[2], Katia Karalis[1], Giusy Della Gatta[1], Tooraj Mirshahi[4], George D. Yancopoulos[2], Olle Melander[5,12], Jonathan Marchini[1], Roberto Tapia-Conyer[8,a], Adam E. Locke[1,a], Aris Baras[1,a], Niek Verweij[1,a], Luca A. Lotta[1,a]

[1]Regeneron Genetics Center, Regeneron Pharmaceuticals Inc, Tarrytown, NY, USA, [2]Regeneron Pharmaceuticals Inc, Tarrytown, NY, USA, [3]Department of Molecular and Functional Genomics, Geisinger Health System, Danville, PA, USA, [4]Geisinger Obesity Institute, Geisinger Health System, Danville, PA, USA, [5]Department of Clinical Sciences Malmö, Lund University, Malmö, Sweden, [6]Department of Medicine, University of Verona, Verona, Italy, [7]Unidad de Medicina Experimental de la Facultad de Medicina de la Universidad Nacional Autónoma de México, Mexico City, Mexico, [8]Instituto Tecnológico y de Estudios Superiores de Monterrey, Monterrey, Mexico, [9]MRC Population Health Research Unit, Nuffield Department of Population Health, University of Oxford, Oxford, UK, [10]Clinical Trial Service Unit & Epidemiological Studies Unit Nuffield Department of Population Health, University of Oxford, Oxford, UK, [11]Department of Genetics, Perelman School of Medicine, University of Pennsylvania, Philadelphia, PA, USA, [12]Department of Emergency and Internal Medicine, Skåne University Hospital, Malmö, Sweden.

Correspondence to: luca.lotta@regeneron.com and aris.baras@regeneron.com
[a] These authors contributed equally.

This file includes:
Supplementary Results
Supplementary Figures 1 to 21
References

Other Supplementary Material for this manuscript includes the following:
Supplementary Data 1 to 28 (.xlsx)

Regeneron Genetics Center

Parsa Akbari, Olukayode A. Sosina, Jonas Bovijn, Jonas B. Nielsen, Minhee Kim, Senem Aykul, Tanima De, Mary E. Haas, George Hindy, Nan Lin, Benjamin Geraghty, Marcus Jones, Michelle G. LeBlanc, Suganthi Balasubramanian, John D. Overton, Jeffrey G. Reid, Alan R. Shuldiner, Michael Cantor, Goncalo R. Abecasis, Manuel A. R. Ferreira, Aris N. Economides, Katia Karalis, Giusy Della Gatta, Jonathan Marchini, Adam E. Locke, Aris Baras, Niek Verweij, Luca A. Lotta, Giovanni Coppola, Katherine Siminovitch, Christina Beechert, Caitlin Forsythe, Erin D. Fuller, Zhenhua Gu, Michael Lattari, Alexander Lopez, Maria Sotiropoulos Padilla, Manasi Pradhan, Kia Manoochehri, Thomas D. Schleicher, Louis Widom, Sarah E. Wolf, Ricardo H. Ulloa, Amelia Averitt, Nilanjana Banerjee, Dadong Li, Sameer Malhotra, Deepika Sharma, Jeffrey Staples, Xiaodong Bai, Suganthi Balasubramanian, Suying Bao, Boris Boutkov, Siying Chen, Gisu Eom, Lukas Habegger, Alicia Hawes, Shareef Khalid, Olga Krasheninina, Rouel Lanche, Adam J. Mansfield, Evan K. Maxwell, George Mitra, Mona Nafde, Sean O'Keeffe, Max Orelus, Razvan Panea, Tommy Polanco, Ayesha Rasool, William Salerno, Jeffrey C. Staples, Kathie Sun, Jiwen Xin, Joshua Backman, Amy Damask, Lee Dobbyn, Arkopravo Ghosh, Lauren Gurski, Eric Jorgenson, Michael Kessler, Jack Kosmicki, Alexander Li, Nan Lin, Daren Liu, Anthony Marcketta, Arden Moscati, Carlo Sidore, Eli Stahl, Kyoko Watanabe, Bin Ye, Blair Zhang, Andrey Ziyatdinov, Ariane Ayer, Aysegul Guvenek, Jan Freudenberg, Julie Horowitz, Katherine Siminovitch, Kavita Praveen, Manav Kapoor, Moeen Riaz, Priyanka Nakka, Sahar Gelfman, Sujit Gokhale, Veera Rajagopal, Bin Ye, Gannie Tzoneva, Juan Rodriguez-Flores, Shek Man Chim, Valerio Donato, Daniel Fernandez, Alessandro Di Gioia, Kristen Howell, Lori Khrimian, Hector Martinez, Lawrence Miloscio, Sheilyn Nunez, Elias Pavlopoulos, Trikaldarshi Persaud, Esteban Chen, Jason Mighty, Lyndon J. Mitnaul, Nirupama Nishtala, Nadia Rana.

DiscovEHR Collaboration

Lance J. Adams, Jackie Blank, Dale Bodian, Derek Boris, Adam Buchanan, David J. Carey, Ryan D. Colonie, F. Daniel Davis, Ian R. Dinsmore, Dustin N. Hartzel, Melissa Kelly, H. Lester Kirchner, Joseph B. Leader, Jonathan Z. Luo, David H. Ledbetter, J. Neil Manus, Christa L. Martin, Michelle Meyer, Tooraj Mirshahi, Matthew Oetjens, Thomas Nate Person, Christopher D. Still, Natasha Strande, Amy Sturm, Jen Wagner, Marc Williams.

Contents

SUPPLEMENTARY RESULTS 37

Supplementary Result 1. Association of pLOF variants in *PLIN1* with favorable fat distribution. 37

Supplementary Result 2. Genomic context analyses for rare variants at the *INHBE* locus...... 38

Supplementary Result 3. Rare pLOF variants in *INHBE* are not associated with estimated bone mineral density or fracture risk, while rare coding variants in *PPARG* show an association... 39

Supplementary Result 4. Interplay of common and rare alleles in body fat distribution.......... 40

SUPPLEMENTARY FIGURES 41

Supplementary Figure 1. Genome- and exome wide associations with BMI-adjusted WHR.. 41

Supplementary Figure 2. Correlations of association estimates in sensitivity analyses using alternative adjustments. 42

Supplementary Figure 3. Associations with magnetic resonance imaging (MRI) derived visceral to gluteofemoral fat ratio for genes identified in the BMI-adjusted WHR discovery analysis. 43

Supplementary Figure 4. Tissue expression enrichment in the exome-wide gene-burden analysis. 44

Supplementary Figure 5. Tissue expression for each of the 16 genes in this study................. 45

Supplementary Figure 6. Association of rare pLOF variants in *INHBE* with bioelectrical impedance body composition measures. 46

Supplementary Figure 7. Associations with BMI-adjusted WHR of common variants at the *INHBE* locus. 47

Supplementary Figure 8. *In vitro* expression of the *INHBE* c.299-1G>C splice acceptor variant in cell lysates and conditioned media compared to wild-type *INHBE*. 48

Supplementary Figure 9. Associations of rare coding variants in *INHBE*, *PLIN1*, *PDE3B* and *ACVR1C* with fat distribution and risk of type 2 diabetes. 49

Supplementary Figure 10. Liver mRNA expression of *INHBE* is upregulated in bariatric surgery patients with histopathologic alterations at liver biopsy. 50

Supplementary Figure 11. Correlation of hepatic *INHBE* mRNA expression with expression of *FST*, *INHBA*, and *INHBB*. 51

Supplementary Figure 12. Phenotypic relationships with liver traits for fat distribution and BMI. 52

Supplementary Figure 13. Prevalence of type 2 diabetes by percentiles of fat distribution and BMI. 53

Supplementary Figure 14. Association of favorable fat distribution polygenic scores with DXA phenotypes. 54

Supplementary Figure 15. Association of polygenic score for lower insulin resistance with liver phenotypes, type 2 diabetes and coronary artery disease risk. 55
Supplementary Figure 16. Model selection step for the generation of a BMI-adjusted WHR polygenic score.. 56
Supplementary Figure 17. Fat distribution, adipose expandability, ectopic liver fat, and risk of type 2 diabetes and liver disease in quantiles of a fat distribution genome-wide polygenic score. 57
Supplementary Figure 18. Association of a polygenic score for higher BMI-adjusted WHR with fat distribution and type 2 diabetes risk. 58
Supplementary Figure 19. Association of a polygenic score for higher BMI-adjusted WHR with refined measures of fat distribution and adipose expandability at DXA. 59
Supplementary Figure 20. Model selection for BMI-adjusted WHR polygenic score in MDCS using GWAS data from a previously published study as the training dataset. 60
Supplementary Figure 21. Rare predicted loss-of-function variants in *PLIN1* identified by exome sequencing and their distribution on the gene. 61
SUPPLEMENTARY REFERENCES 62

SUPPLEMENTARY RESULTS

Supplementary Result 1. Association of pLOF variants in *PLIN1* with favorable fat distribution.

Two frameshift mutations in *PLIN1* have been previously identified to be causal for FPLD type 4, which is characterized by selective lack of gluteofemoral and limb fat and an unfavorable metabolic profile (*1*). In our analysis, *PLIN1* pLOF variants were associated with lower BMI-adjusted WHR and larger hip circumference (**Supplementary Data 27**), indicative of a favorable fat distribution and opposite of what has been observed in FPLD type 4. The two frameshift variants in *PLIN1* that cause FPLD type 4 (Leu404fs and Val398fs) occur at the C-terminal end of the protein, with long out-of-frame C-terminal tails with 158 and 166 aberrant amino acid residues (*1*), and both mutations also result in the synthesis of a mutated protein that is longer than wild-type perilipin 1 (*2*). Conversely, the vast majority of pLOF alleles observed in our data are predicted to truncate perilipin 1 earlier than the two FPLD variants (**Supplementary Figure 21**) and are likely to result in loss of a functional copy of the gene, leading to haploinsufficiency. Therefore, our human genetic analysis suggests that loss of a copy of *PLIN1* is associated with favorable fat distribution in the general population, while the lipodystrophy phenotype observed in FPLD type 4 might be due to altered function of the C-terminal frameshift variants.

Supplementary Result 2. Genomic context analyses for rare variants at the *INHBE* locus.

We explored in depth the genomic context of the associations with BMI-adjusted WHR at the *INHBE* locus. We looked for fine-mapped common variant signals in a 1-Mb window around the *INHBE* gene and found no associated common variants that met genome-wide statistical significance in any of the analyzed populations (**Supplementary Data 2 and Supplementary Figure 7**), indicative of the association being driven exclusively by rare variants.

In a backward-selection analysis of *INHBE* alleles contributing to the pLOF gene-burden association, we found that the association was primarily, but not exclusively driven by the c.299-1G>C splice variant (**Supplementary Data 14**). As c.299-1G>C is in linkage disequilibrium (LD; r^2=0.89) with a rare Ser544Asn missense variant in the *SLC26A10* pseudogene, we explored the possible role of the latter in the observed associations.

First, despite a lower allele frequency, *INHBE* c.299-1G>C had a stronger association with BMI-adjusted WHR than *SLC26A10*-Ser544Asn and was the lead rare coding variant at the locus (**Supplementary Data 10**). Second, the association for *SLC26A10*-Ser544Asn was eliminated when adjusting for *INHBE* c.299-1G>C genotype (**Supplementary Data 10**). Third, even when excluding all carriers of *SLC26A10*-Ser544Asn from the analysis, which (due to LD) meant the exclusion of the majority of *INHBE* pLOF carriers, there was still evidence of association for rare coding variants in *INHBE* (**Supplementary Data 15**). Consistent with the contribution of *INHBE* rare coding alleles beyond c.299-1G>C, we observed stronger associations for the overall burden of pLOF variants in *INHBE* than the splice variant alone for several WHR-related metabolic traits, including hip circumference, HbA1c, apolipoprotein B, high-density lipoprotein cholesterol, triglycerides and type 2 diabetes (**Supplementary Data 11**). Fourth, when excluding the Ser544Asn allele, rare coding variants in *SLC26A10* were not associated with BMI-adjusted WHR (**Supplementary Data 16**). Fifth, *SLC26A10* is a pseudogene that shows a high degree of tolerance to deleterious rare coding variation (https://gnomad.broadinstitute.org/, accessed January 18th 2021) (*3*), with no reported evidence of protein expression in the Human Protein Atlas (https://www.proteinatlas.org/, accessed January 18th 2021) (*4*). Thus, beyond the association results above, a missense allele in this pseudogene is less likely to impact a human phenotype than an experimentally validated loss-of-function allele like c.299-1G>C in a gene with evidence of protein expression as *INHBE* (https://www.proteinatlas.org/, accessed January 18th 2021) (*4*). Taken together, these results implicate *INHBE* as the effector gene for rare variant associations at the locus.

Supplementary Result 3. Rare pLOF variants in *INHBE* are not associated with estimated bone mineral density or fracture risk, while rare coding variants in *PPARG* show an association.

Peroxisome proliferator activated receptor gamma (PPARG) agonists, a class of anti-diabetic drugs that enhance glycemic control by promoting peripheral fat storage (*5*), cause an increased risk of fractures (*6*), which has partly contributed to their limited clinical use. Consistently, we found an association with lower odds of fractures for rare pLOF plus deleterious missense variants in *PPARG* (**Supplementary Data 28**), which were also associated with higher BMI-adjusted WHR at exome-wide statistical significance in our study (**Table 1**). In contrast, rare pLOF variants in *INHBE* were not associated with estimated bone mineral density or fracture risk (**Supplementary Data 28**).

Supplementary Result 4. Interplay of common and rare alleles in body fat distribution.

We sought to compare phenotypic impact of polygenic extremes and rare coding variants or other genotype combinations. We used protein-truncating or experimentally validated LOF variants in *PPARG* (**Methods**), the causal gene for FPLD type 3 and one of the 16 genes from our exome-wide analysis, as a benchmark for a Mendelian-like effect on fat distribution. Then, using GWAS results, we generated, selected, and validated a polygenic score comprising 500 common variants that maximized the variance explained in BMI-adjusted WHR (**Methods; Supplementary Figure 16**).

PPARG mutation carriers had 0.46 SD higher BMI-adjusted WHR (p=0.012; **Table 2, Table S23**) and >4-fold higher odds of type 2 diabetes (per-allele odds ratio, 4.3; 95% CI, 1.9 to 9.6; $p=3.4\times10^{-4}$; **Table 2, Supplementary Data 23**) compared to noncarriers; effect sizes which are consistent in magnitude to those of other Mendelian-disease causing mutations in population-based studies (*7-9*). In the same dataset, higher values of the 500-variant polygenic score for BMI-adjusted WHR were associated with an unfavorable fat distribution and a higher diabetes risk, with a graded dose-response relationship (**Supplementary Figures 17-19**). Individuals in the top 1% of polygenic predisposition had similar average fat distribution to *PPARG* mutant carriers (**Table 2**). Notably, being in the top 1% of the polygenic score is approximately 120-times more frequent than being the heterozygous carrier of a *PPARG* mutation in the cohorts we studied (**Table 2**).

We observed similarly large phenotypic impact for other genotype combinations including rare alleles combined with high polygenic burden or multiple rare alleles (see **Methods** for genotype selection). Heterozygous carriers of *ANKRD12* pLOF variants (the largest-effect WHR increasing association in our analysis; **Table 1**) who were also in the top quintile of the polygenic score had an average BMI-adjusted WHR of 0.77 SD units, a comparable phenotype to that of *PPARG* mutations, for a genotype combination twice as frequent (**Supplementary Data 24**). Rare "human knock-out" individuals who carried homozygous pLOF variants in *PLIN4*, encoding a coating protein of unilocular lipid droplets in adipocytes, had an average BMI-adjusted WHR of 0.52 SD units (**Supplementary Data 24**), again similar to that of *PPARG* mutation carriers. Interestingly, while the phenotypic impact on fat distribution of those genotypes was similar to that of *PPARG* mutations and carriers of those genotypes had higher prevalence of type 2 diabetes than the population prevalence (**Supplementary Data 24**), the prevalence of diabetes was highest in *PPARG* mutation carriers (33%; **Supplementary Data 24**) suggesting that perhaps the impact of *PPARG* mutations on diabetes is only partly mediated via fat distribution.

We made similar observations at the opposite polygenic extreme. Individuals in lower quantiles of the BMI-adjusted WHR polygenic score had more favorable fat distribution and lower prevalence of metabolic disease (**Supplementary Figures 17-19**), with individuals at the bottom 1% of the polygenic score having a favorable fat distribution (-0.67 SDs) and a risk of type 2 diabetes similar to that of *INHBE* pLOF carriers (**Table 2**). Interestingly, individuals with rare pLOF variants in *INHBE* who were also in the bottom quintile of the polygenic score had a prevalence of diabetes of just 1% (as opposed to a pooled prevalence of 9.8% in the studied cohorts; **Supplementary Data 24**).

Our inferences remained the same when polygenic scores were derived using a previously-published GWAS training set of 142,762 people (*10*) (**Supplementary Data 25, Supplementary Figure 20**).

SUPPLEMENTARY FIGURES

Supplementary Figure 1. Genome- and exome wide associations with BMI-adjusted WHR.

Manhattan plots showing statistical strength of association (y-axis; plotted as -log10(p-value)) and chromosome position (x-axis). Panel A shows the multi-ancestry exome-wide analysis of gene burden genotypes; Panel B the multi-ancestry exome-wide analysis of single rare coding variants; Panel C shows the European Ancestry and Panel D shows the American Ancestry genome-wide association analysis of common imputed variants. P-values are from two-sided Z-tests from fixed-effect meta-analyses. For the exome-wide analyses, triangles pointing upwards (in yellow) or downwards (red) indicate associations with higher and lower BMI-adjusted WHR, respectively. Abbreviations: log, logarithm.

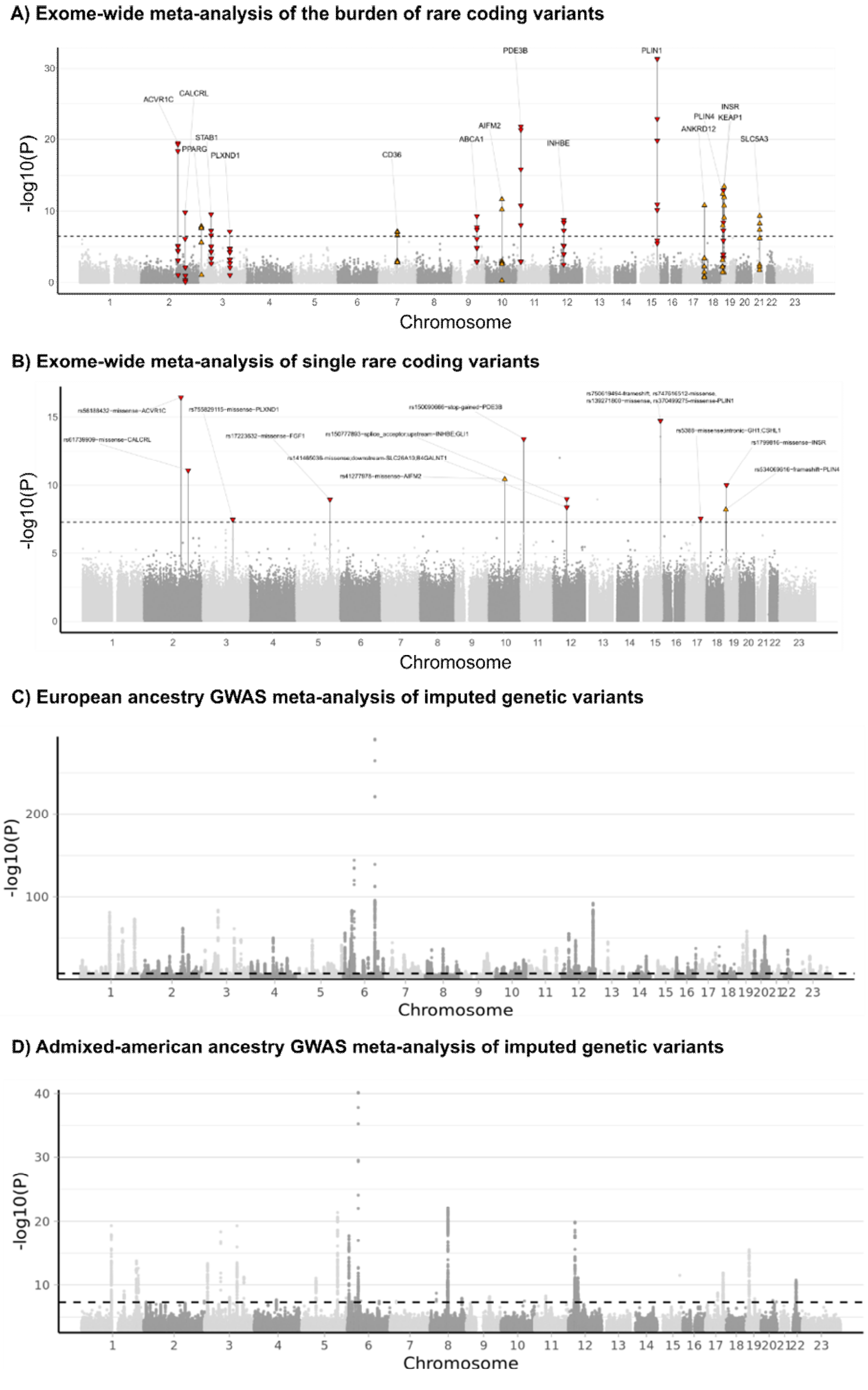

Supplementary Figure 2. Correlations of association estimates in sensitivity analyses using alternative adjustments.

Each panel shows associations for the 16 genes identified in the BMI-adjusted WHR discovery analysis, with BMI-adjusted WHR (x-axis; n=618,375) and with the following trait associations on the y-axis: A, WHR, not adjusted for BMI (n=619,298); B, WHR, adjusted for BMI and height (n=618,375); C, WHR, adjusted for BMI and estimated bone mineral density (n=440,770); D, WHR, non-linear adjusted for BMI and total body fat mass (n=444,107). Non-linear adjustment (panel D) was performed using a cubic spline. Error bars represent 95% confidence intervals around the beta coefficient. The red dotted line corresponds to a line with a slope of 1 and intercept of zero; the solid blue line represents the ordinary least squares regression line. P-values are based on two-sided Wald tests. Abbreviations: eBMD, estimated bone mineral density; SD, standard deviation; CI, confidence interval; WHR, waist to hip ratio; BMI, body mass index; UKB, UK Biobank.

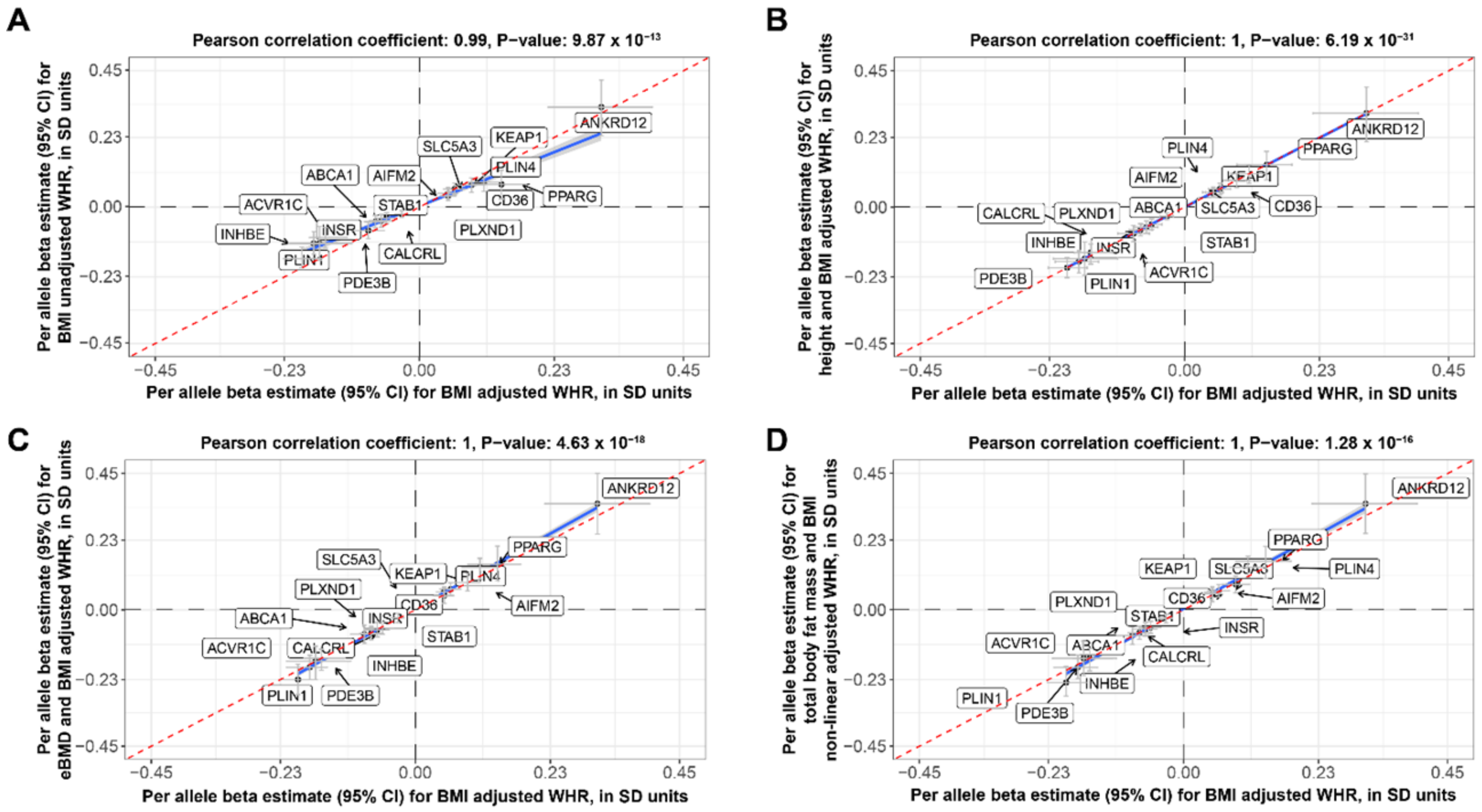

Supplementary Figure 3. Associations with magnetic resonance imaging (MRI) derived visceral to gluteofemoral fat ratio for genes identified in the BMI-adjusted WHR discovery analysis.

The Figure shows associations with MRI-derived visceral to gluteofemoral fat ratio (VA/G fat ratio; y-axis; n=38,878; see **Methods**) and BMI-adjusted WHR (x-axis; n=618,375) for the 16 genes identified in the discovery analysis. Error bars represent 95% confidence intervals around the beta coefficient (black dots) in SD units of the outcome trait per-allele. The dotted line corresponds to the null effect of zero; the solid black line shows the Huber weighted robust regression line. P-value is from a two-sided Wald test. Abbreviations: SD, standard deviation; CI, confidence interval; WHR, waist to hip ratio; BMI, body mass index.

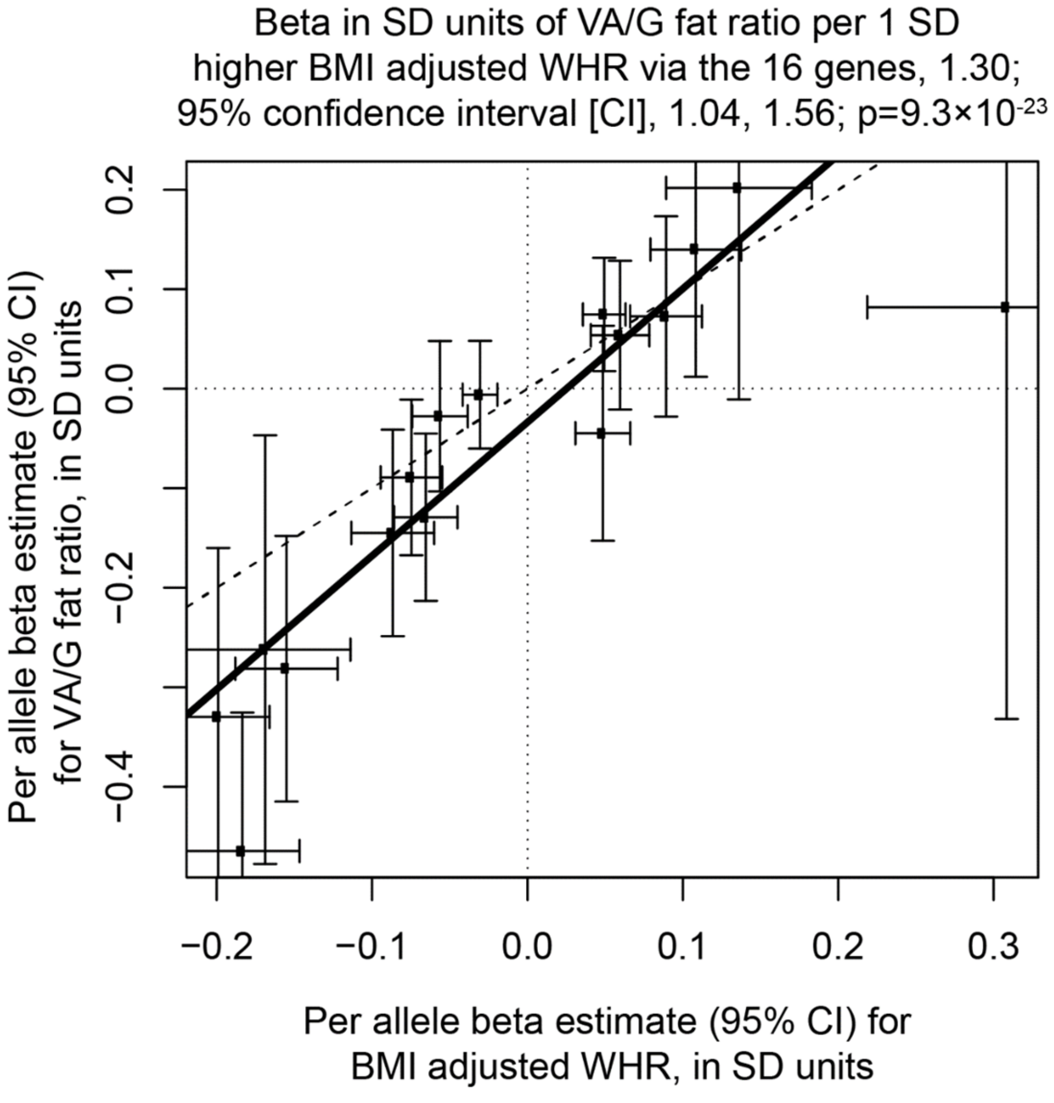

Supplementary Figure 4. Tissue expression enrichment in the exome-wide gene-burden analysis.

Tissue enrichment analysis was performed using both GTEx V8 gene expression data and gene-burden association analysis results for BMI-adjusted WHR. Shown on the plot are tissues (y-axis) whose enhanced genes (see **Methods**) have a stronger association (x-axis) with BMI-adjusted WHR. Subcutaneous adipose tissue was preferentially sampled from the leg (*11*). P-values are from one-sided Wald tests. Only adipose subcutaneous tissue showed significant enrichment at α=0.001 (i.e. a Bonferroni correction for 48 tissues tested for enrichment). Abbreviations: p, P-value.

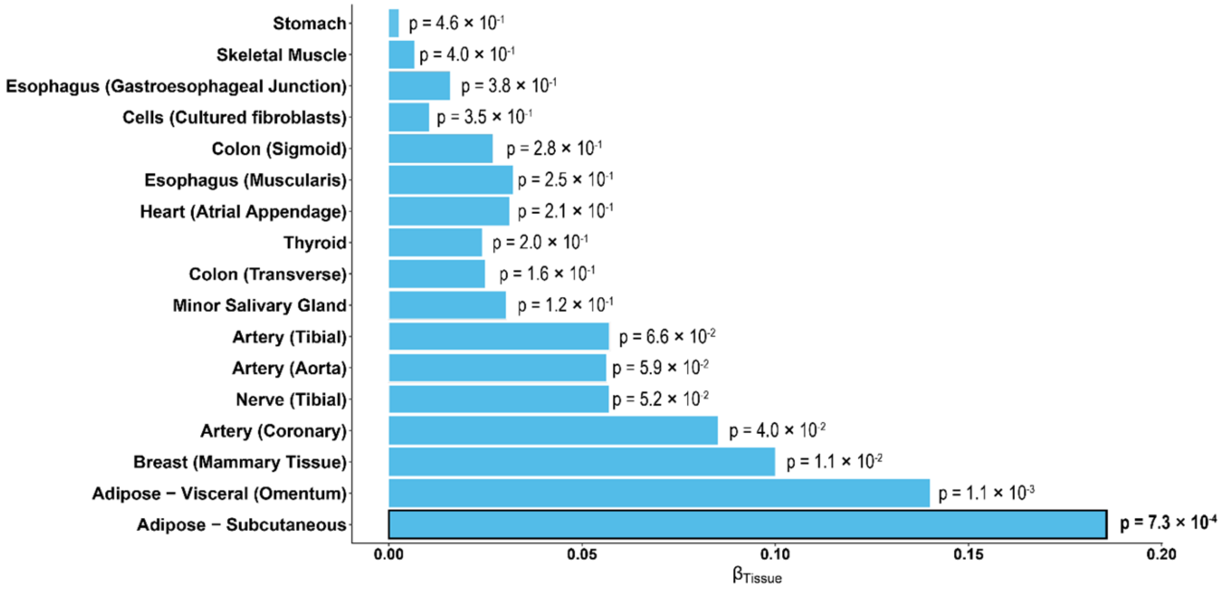

Supplementary Figure 5. Tissue expression for each of the 16 genes in this study.

We identified tissues of enriched expression for each of the 16 genes identified in our study. The figure below shows, for a given gene, the enriched tissue where that gene is most expressed (see **Methods**). Subcutaneous adipose tissue was preferentially sampled from the leg (*11*).

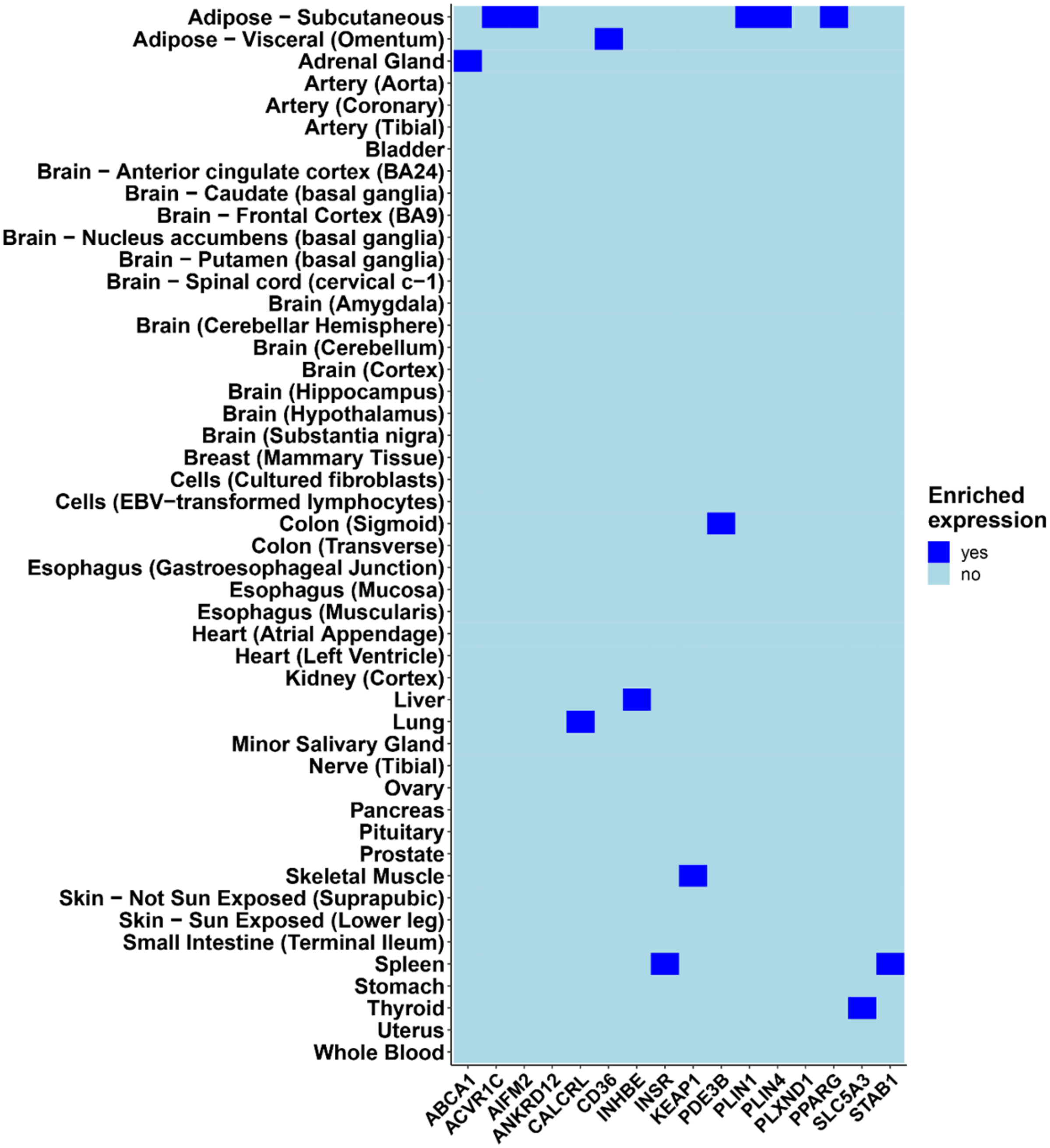

Supplementary Figure 6. Association of rare pLOF variants in *INHBE* with bioelectrical impedance body composition measures.
Association estimates are represented in red for fat mass or percentage phenotypes and in blue for lean mass or percentage phenotypes. Markers represent beta coefficients and error bars their 95% confidence intervals. Data are from the UKB cohort (n=445,122). Abbreviations: pLOF, predicted loss of function; AAF, alternative allele frequency; kg, Kilograms; CI, confidence intervals.

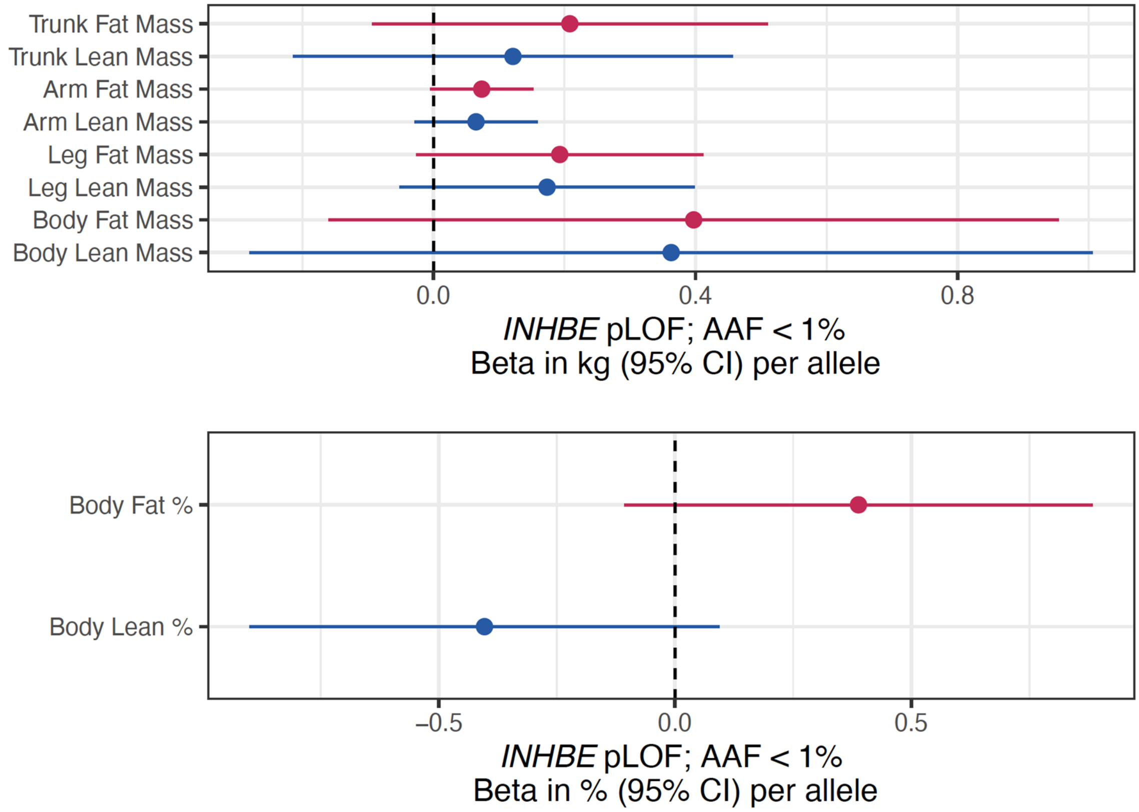

Supplementary Figure 7. Associations with BMI-adjusted WHR of common variants at the *INHBE* locus.

GWAS analyses of common imputed variants in European ancestry individuals from UKB and MDCS (n=513,838) (Panel A) and in American ancestry individuals from MCPS (n=138,188) (Panel B) show there are no variants reaching genome-wide significance threshold ($p<5x10^{-8}$, $-\log_{10}(p) > 7.30$) within a 1-Megabase window around *INHBE* (shown in red). Each blue circle is a genetic variant. P-values are based on two-sided Wald tests. Abbreviations: log, logarithm; BMI, body mass index; WHR, waist-hip ratio; UKB, UK Biobank; MDCS, Malmo Diet and Cancer Study; MCPS, Mexico City Prospective Study.

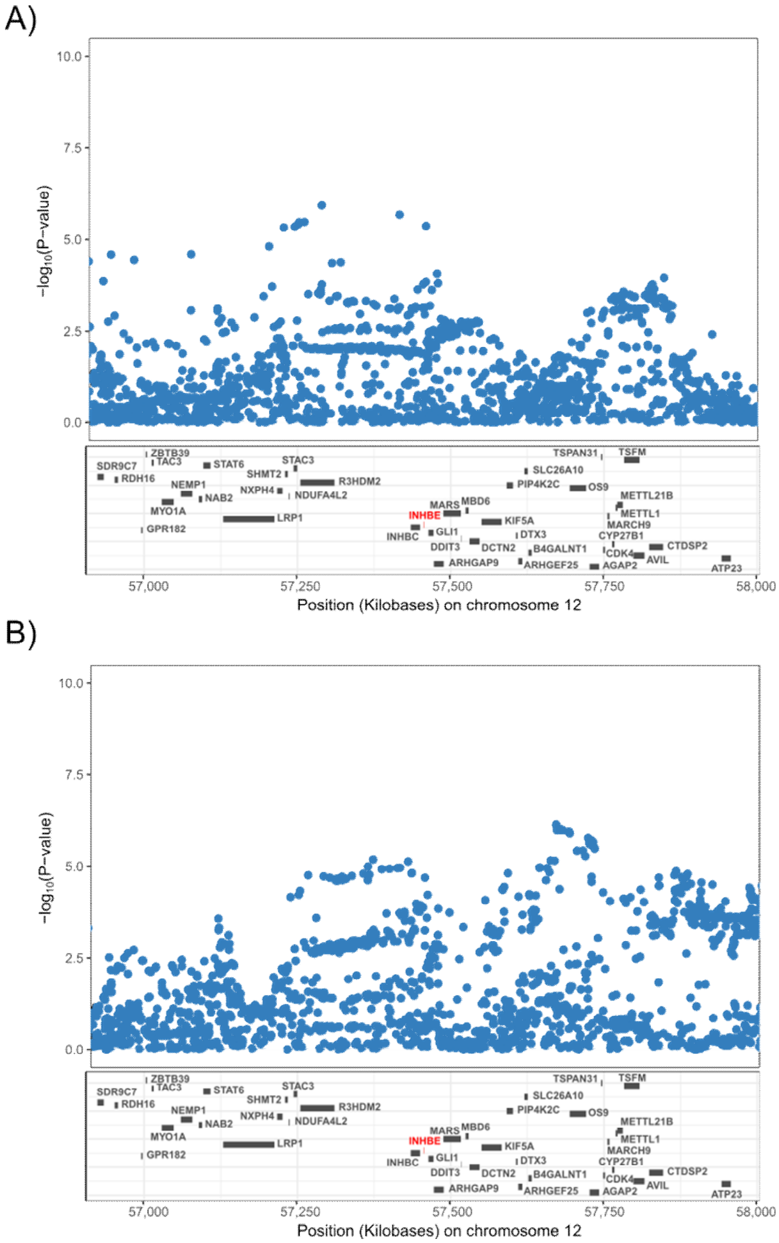

Supplementary Figure 8. *In vitro* expression of the *INHBE* c.299-1G>C splice acceptor variant in cell lysates and conditioned media compared to wild-type *INHBE*.

Western blot analysis detecting INHBE protein in cell lysates (left-hand panel, with blue header) and conditioned media (right-hand panel, with green header) from CHO cells transfected with wild type *INHBE* or the *INHBE* predicted loss of function splice acceptor (c.299-1G>C) variant. Full length GST-tagged recombinant INHBE protein (100ng) was used as a positive control; complete Ponceau S stains for comparison of sample loading across lanes are shown. The image is a representative image of one of three technical replicates. Each replicate yielded similar results. Abbreviations: CHO, Chinese hamster ovary; GST, Glutathione s-transferase; WT, wildtype; WB, western blot; Ponceau S, Ponceau S (Acid Red 112); CM, conditioned media; kDa, kilodalton

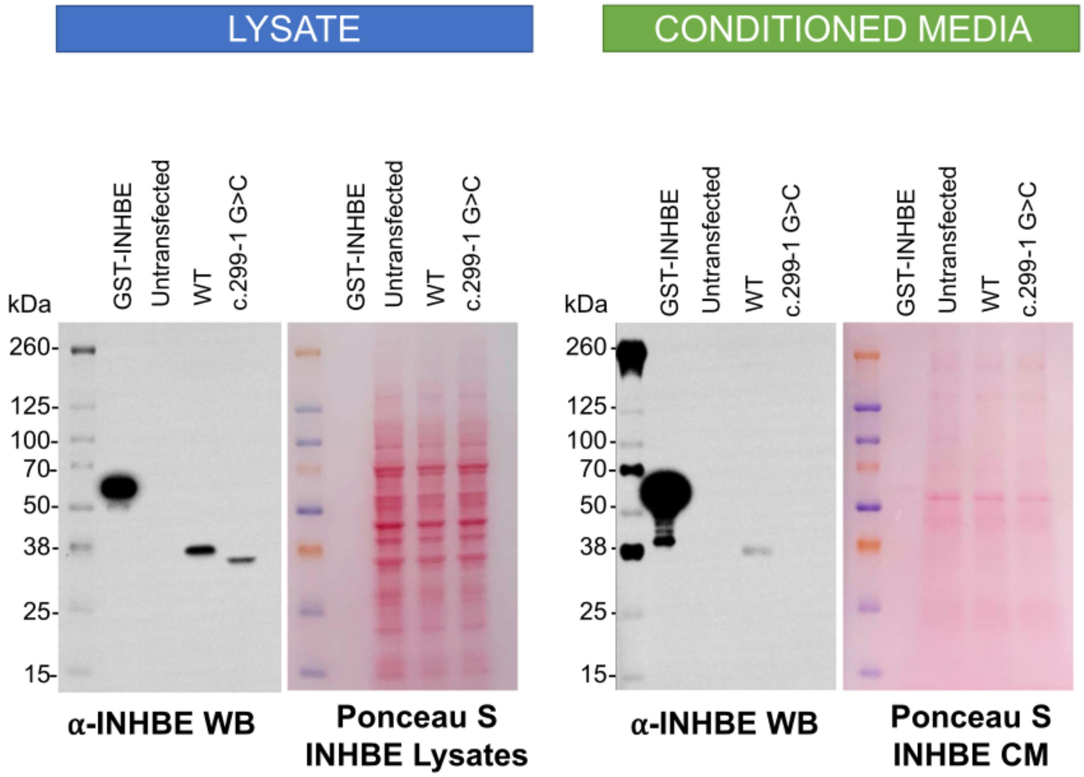

Supplementary Figure 9. Associations of rare coding variants in *INHBE*, *PLIN1*, *PDE3B* and *ACVR1C* with fat distribution and risk of type 2 diabetes.

Genes were included in this analysis if they had a large-effect association with lower BMI-adjusted WHR in our discovery analysis (i.e. gene-burden beta lower than -0.1 SD units per-allele). Estimates for the association with BMI-adjusted WHR are on the x-axis (n=618,375), and estimates for the association with type 2 diabetes are on the y-axis (92,205 cases and 610,914 controls). Markers represent the point estimate (beta coefficient and log-odds ratio) and error bars their 95% confidence intervals. Abbreviations: SD, standard deviation; CI, confidence interval.

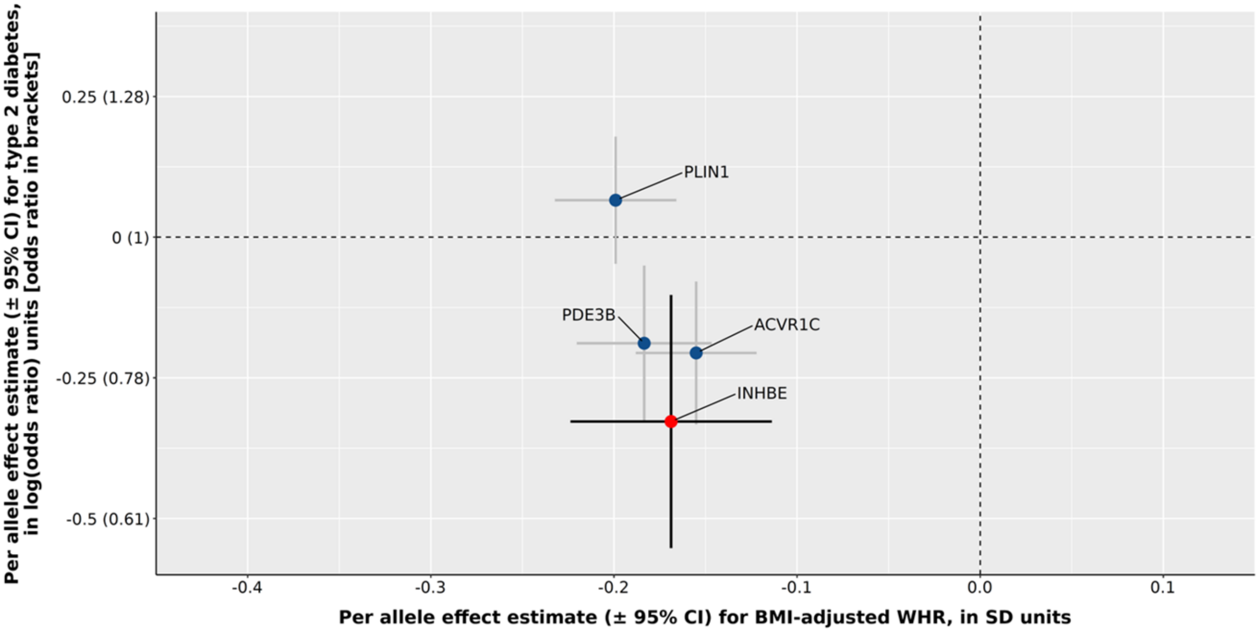

Supplementary Figure 10. Liver mRNA expression of *INHBE* is upregulated in bariatric surgery patients with histopathologic alterations at liver biopsy.

In the top panel, the figure shows liver mRNA expression levels of *INHBE* in counts per million (CPM) in patients (n=2,032) with normal liver (control), steatosis of the liver (simple steatosis) and nonalcoholic steatohepatitis (NASH). The simple steatosis group showed higher expression of *INHBE* in the liver than the control group. The NASH group showed higher expression both when compared to the control and when compared to the simple steatosis groups. Box plots depict the median (horizontal bar), the 75th and 25th percentiles respectively (top and bottom bounds of each box), and the minimum and maximum CPM values for *INHBE*. In the bottom panel, the figure shows differential expression results comparing individuals in each nonalcoholic fatty liver disease (NAFLD) activity score (NAS) group compared with individuals with a score of zero. The numbers above each point correspond to the sample size in each group. All differences in *INHBE* expression between groups were statistically significant after correction for multiple testing. Markers represent the estimated percentage change and error bars their 95% confidence intervals. P-values are based on two-sided Wald tests.

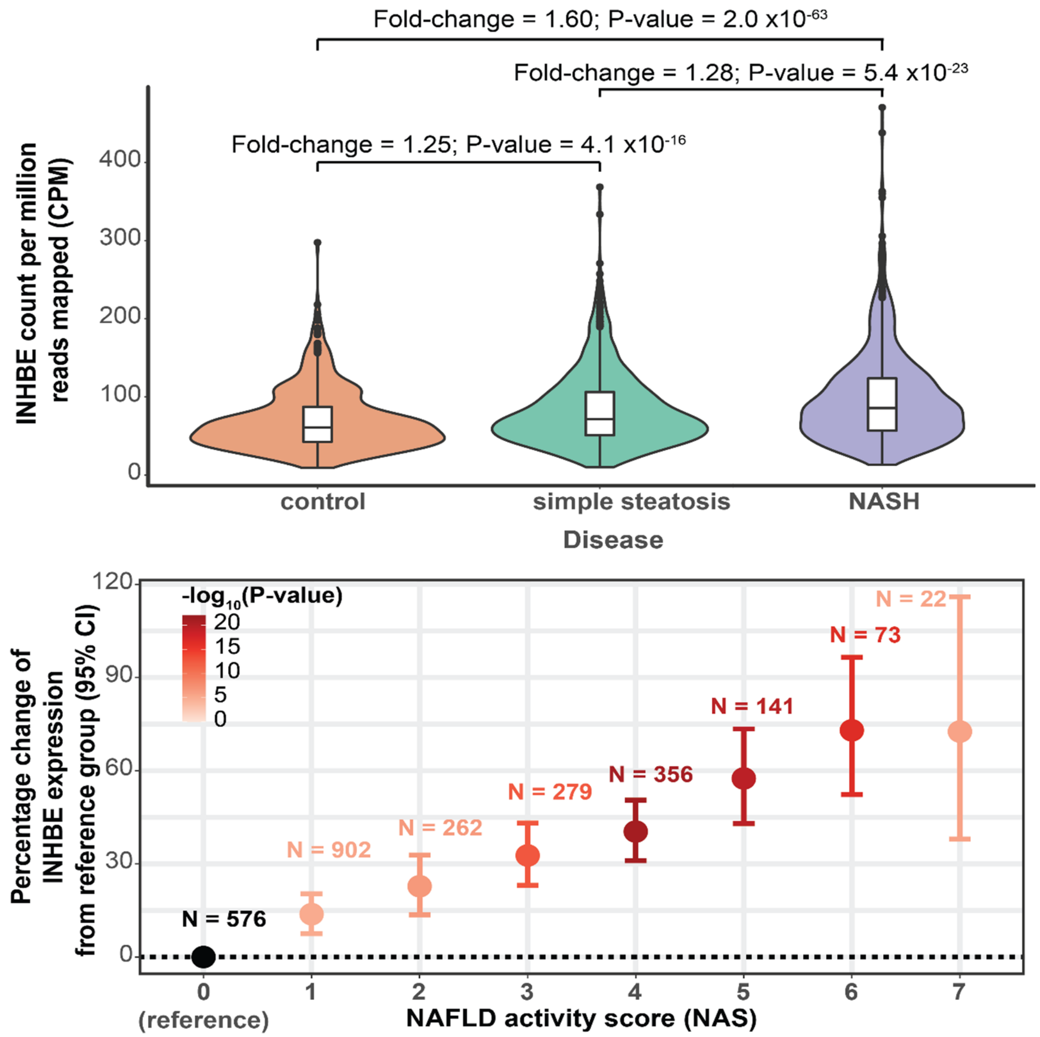

Supplementary Figure 11. Correlation of hepatic *INHBE* mRNA expression with expression of *FST*, *INHBA*, and *INHBB*.

The figure shows the correlation (estimated using Pearson's correlation coefficient, with 95% CI in parenthesis) between liver mRNA expression levels of *INHBE* (shown on the y-axis), and liver expression of *FST* (encoding follistatin), and *INHBA* and *INHBB* (encoding the activin subunits; shown on the x-axis).

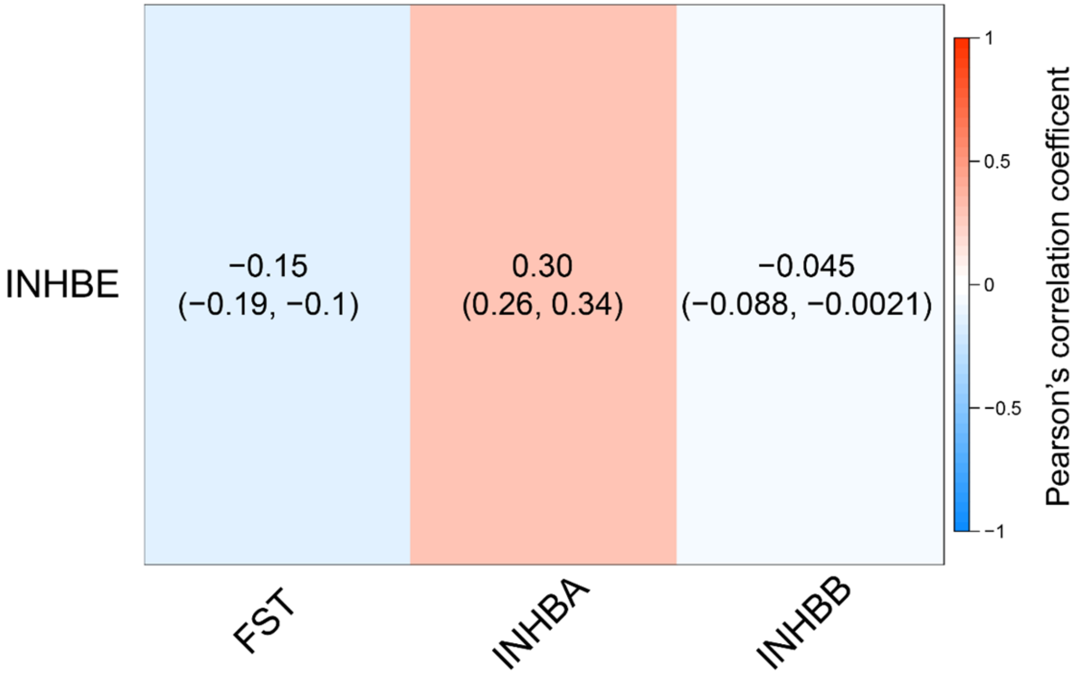

Supplementary Figure 12. Phenotypic relationships with liver traits for fat distribution and BMI.

The figure below shows the relationships of BMI-adjusted WHR (green points) or BMI (orange points) with alanine aminotransferase (ALT), aspartate aminotransferase (AST), magnetic resonance imaging derived proton density liver fat fraction (PDFF; a measure of liver fat percentage), magnetic resonance imaging derived iron-corrected T1 mapping (cT1; a measure of liver inflammation), nonalcoholic fatty liver disease (NAFLD) or nonalcoholic steatohepatitis (NASH), and liver cirrhosis. Analyses were performed in European ancestry participants from the UKB. Abbreviations: BMI-adjusted WHR, waist-hip ratio adjusted for body mass index; BMI, body mass index; SD, standard deviation; U/L international units per liter; ms, milliseconds; UKB, UK Biobank.

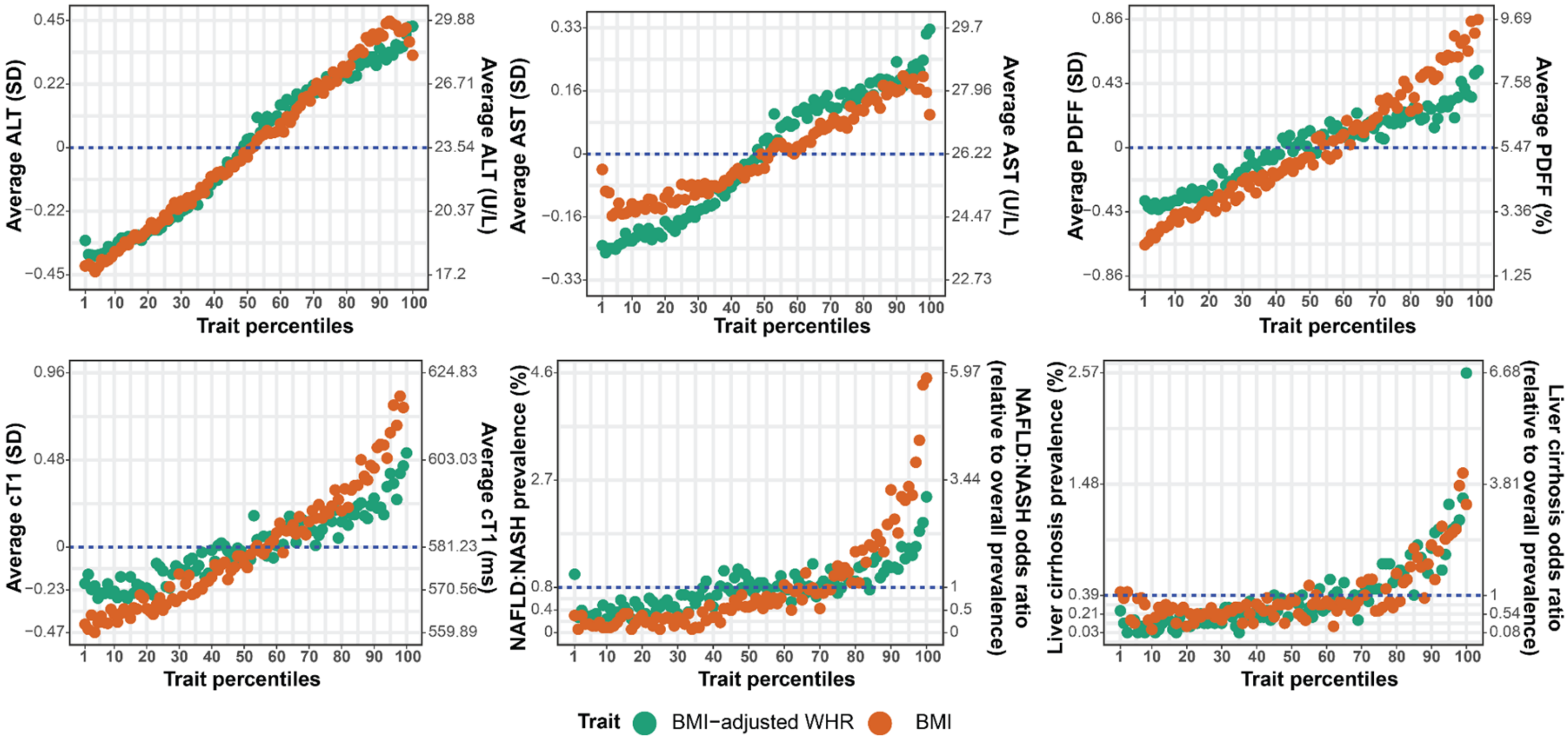

Supplementary Figure 13. Prevalence of type 2 diabetes by percentiles of fat distribution and BMI.

The figure below shows the relationships of BMI-adjusted WHR (green points) and BMI (orange points) with type 2 diabetes. Analyses were performed in European ancestry participants from the UKB. Abbreviations: BMI-adjusted WHR, waist-hip ratio adjusted for body mass index; BMI, body mass index; UKB, UK Biobank.

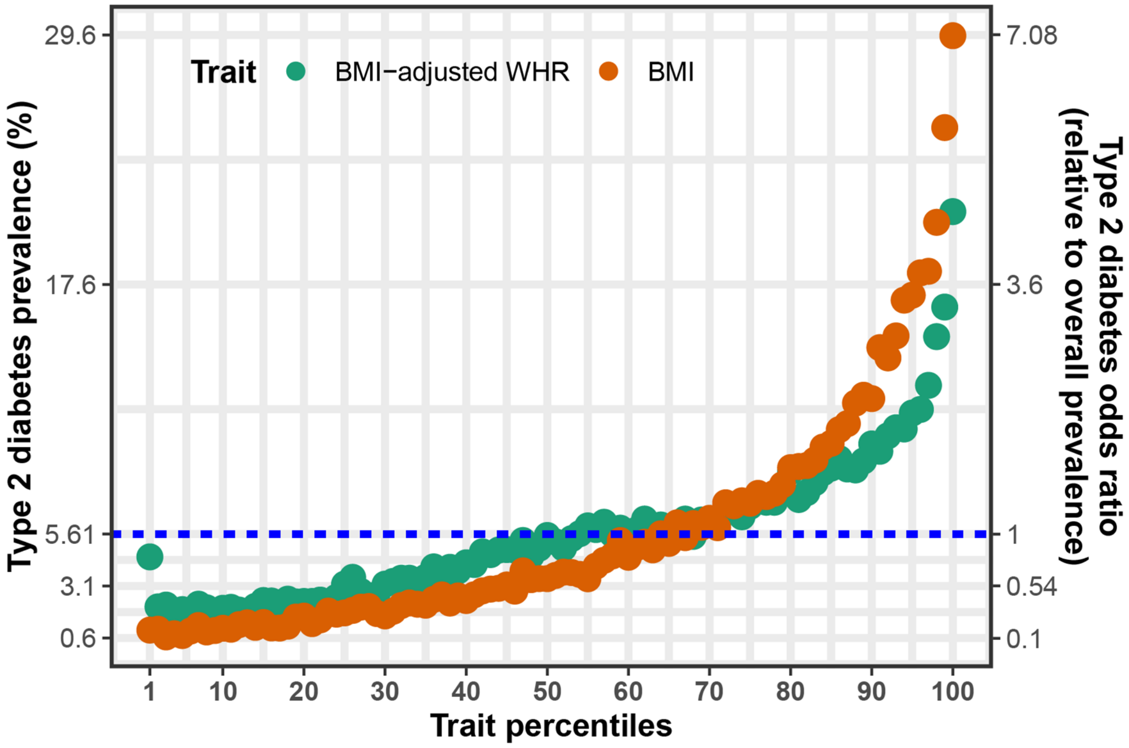

Supplementary Figure 14. Association of favorable fat distribution polygenic scores with DXA phenotypes.

Panel A shows associations with VA/G fat mass ratio (n=4,959), which was adjusted for BMI. Panel B shows associations with leg fat percentage (n=4,966), calculated using total body fat as the denominator. Panel C shows associations with leg fat mass (n=4,966). Associations of a polygenic score for lower BMI are also shown for comparison. Markers represent beta coefficients and error bars their 95% confidence intervals. P-values are based on two-sided Wald tests. Abbreviations: DXA, Dual-energy X-ray absorptiometry; CI, confidence interval; BMI, body mass index; WHR, waist-hip ratio; SD, standard deviation; P, p-value.

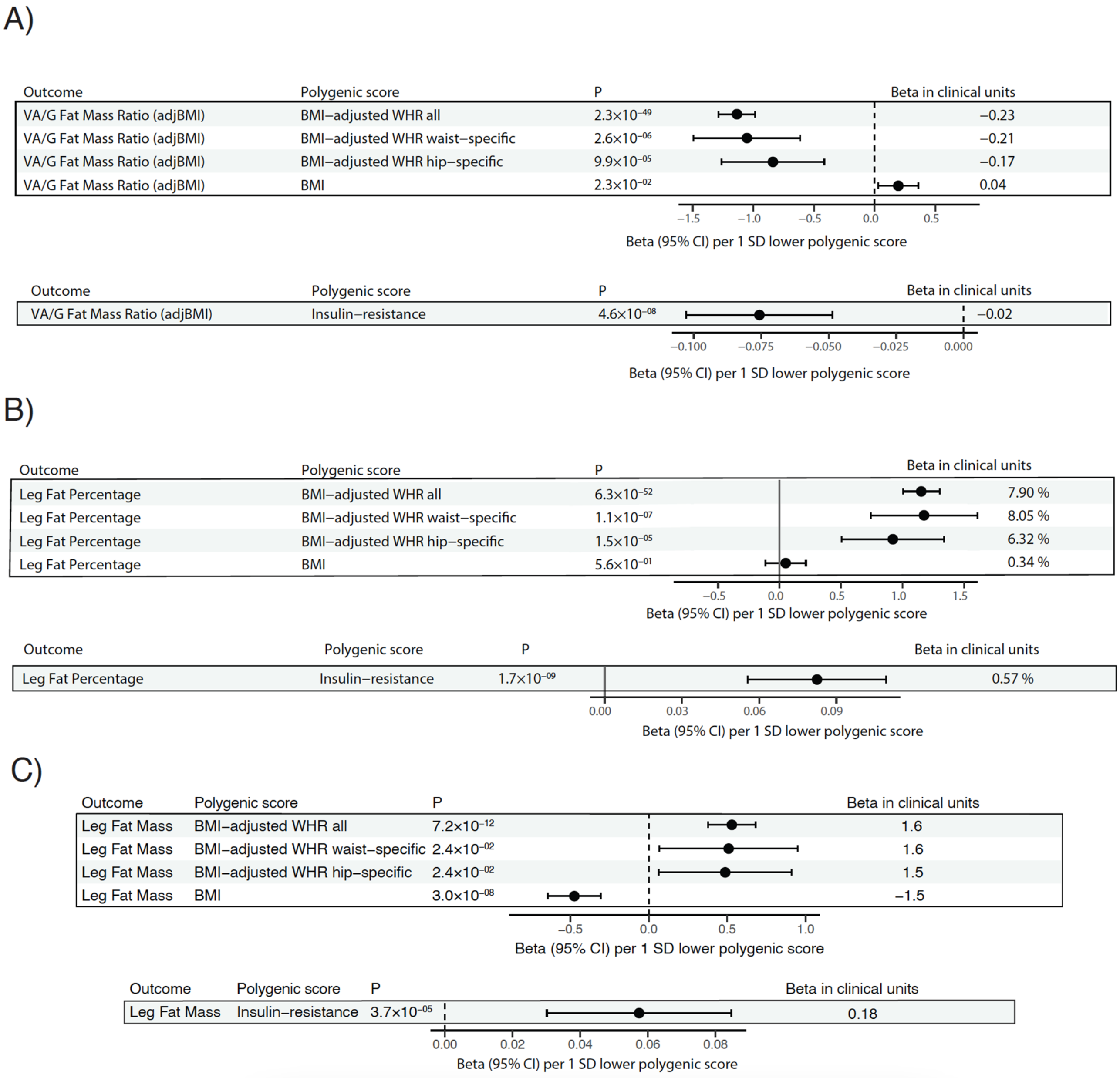

Supplementary Figure 15. Association of polygenic score for lower insulin resistance with liver phenotypes, type 2 diabetes and coronary artery disease risk.

Estimates are shown per 1 SD lower insulin resistance polygenic score. Markers represent beta coefficients (for quantitative traits) or odds ratios (for binary traits) and error bars their 95% confidence intervals. The upper panel shows associations with quantitative traits, while the lower panel associations with binary traits. P-values are based on two-sided Wald tests. Sample size per outcome phenotype: ALT, 442,695; PDFF at MRI imaging, 38,915; cT1 at MRI imaging, 38,915; NAFLD activity score at liver biopsy, 3,572; nonalcoholic liver disease, 14,195 cases and 428,139 controls; cirrhosis, 4,063 cases and 428,139 controls; type 2 diabetes, 58,379 cases and 530,072 controls; coronary artery disease, 89,202 cases and 342,007 controls. Abbreviations: ALT, alanine aminotransferase; AST, aspartate aminotransferase; PDFF, proton density liver fat fraction; cT1, corrected T1; NAFLD, nonalcoholic fatty liver disease; BMI, body mass index; WHR, waist-hip ratio; P, P-value; CI, confidence intervals; SD, standard deviation.

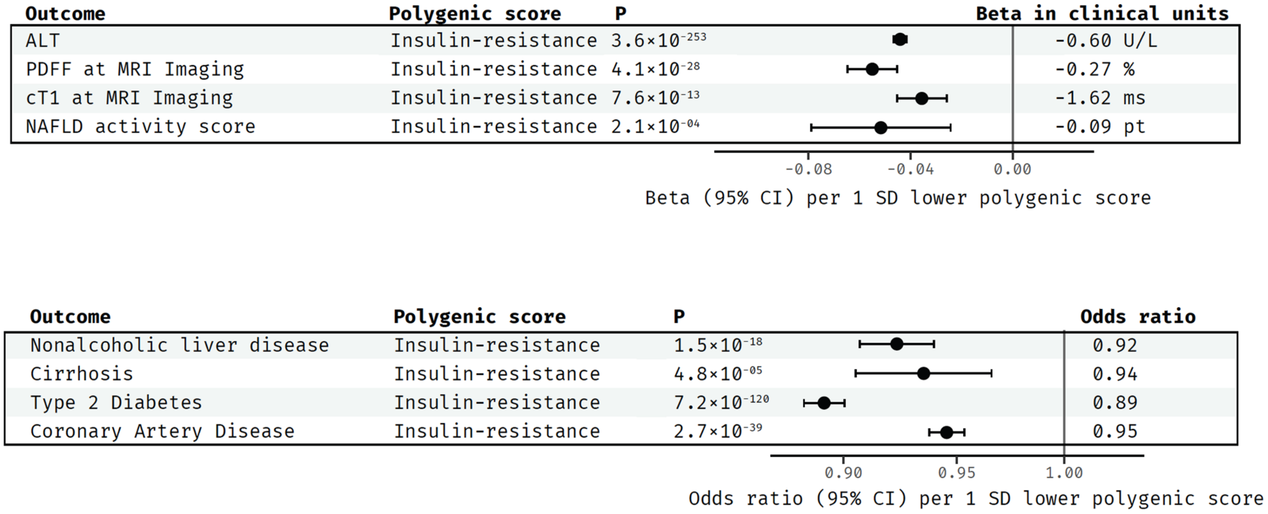

Supplementary Figure 16. Model selection step for the generation of a BMI-adjusted WHR polygenic score.

The plot reports the variance explained in the BMI-adjusted WHR phenotype (top panel) and the number of variants included in the score (bottom panel) for each of the different polygenic score generation approaches. Analyses were performed in the Malmö Diet and Cancer Study cohort. Abbreviations: BMI-adjusted WHR, waist-hip ratio adjusted for body mass index; Cojo, conditional and joint analysis approach; P+T, clumping and thresholding approach; p, P-value threshold used; pi, assumed proportion of non-zero effect variants; R_sqrd, squared Pearson correlation coefficient threshold used to filter variants; R^2, proportion of variance explained in BMI-adjusted WHR by its polygenic score after covariate adjustment (see **Methods**); log, logarithm.

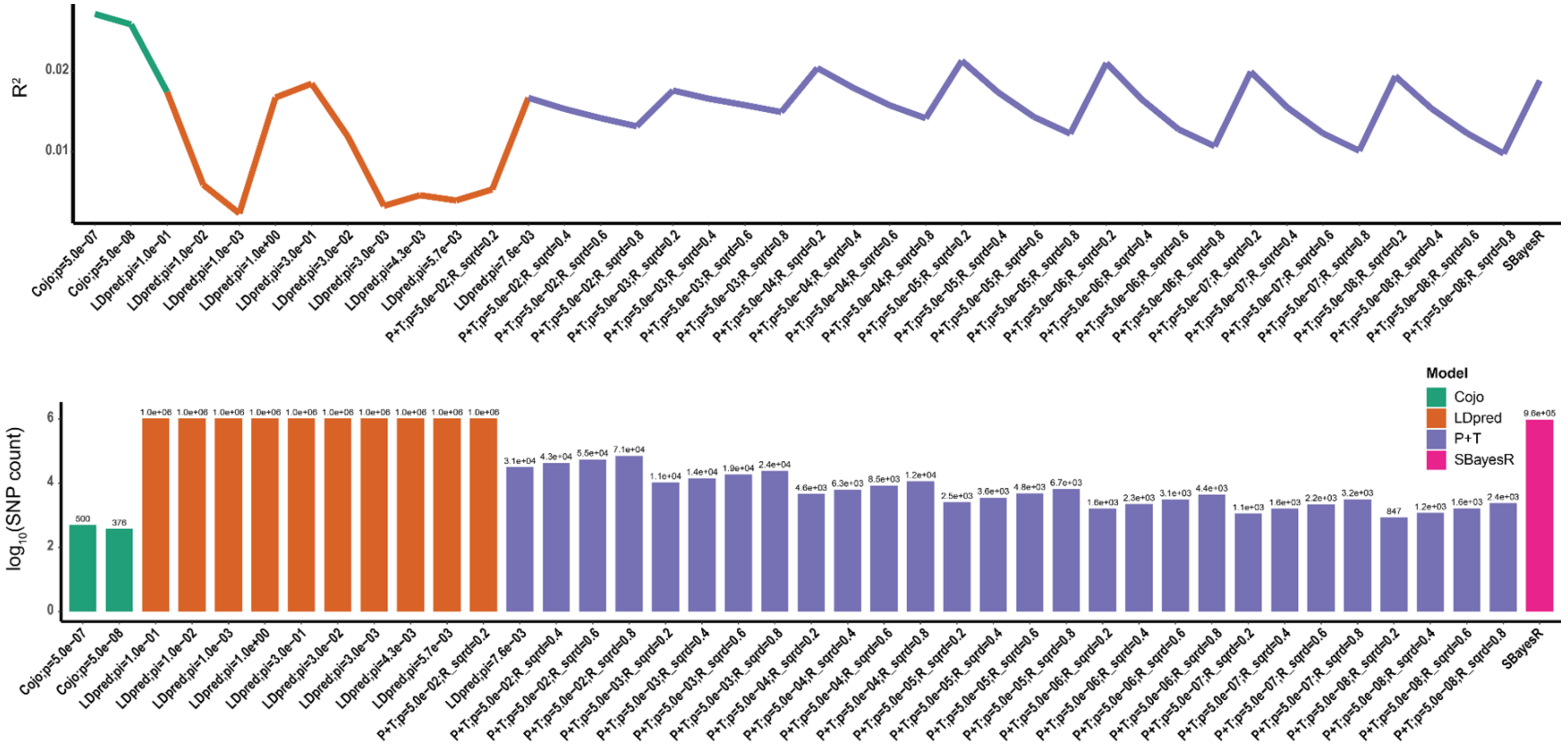

Supplementary Figure 17. Fat distribution, adipose expandability, ectopic liver fat, and risk of type 2 diabetes and liver disease in quantiles of a fat distribution genome-wide polygenic score.

Proton density fat fraction at liver MRI is a measure of the proportion of liver parenchyma occupied by fat. The blue dashed lines correspond to the population mean (for continuous traits) or the overall prevalence (for binary traits). Plots for BMI-adjusted WHR, leg fat percentage of body fat, and proton density fat fraction are based on UKB data, while the plots for type 2 diabetes, non-alcoholic liver disease, and liver cirrhosis are based on data from the UKB and the GHS cohorts. Abbreviations: BMI, body mass index; WHR, waist-hip ratio; SD, standard deviations; MRI, magnetic resonance imaging; NALD, non-alcoholic liver disease.

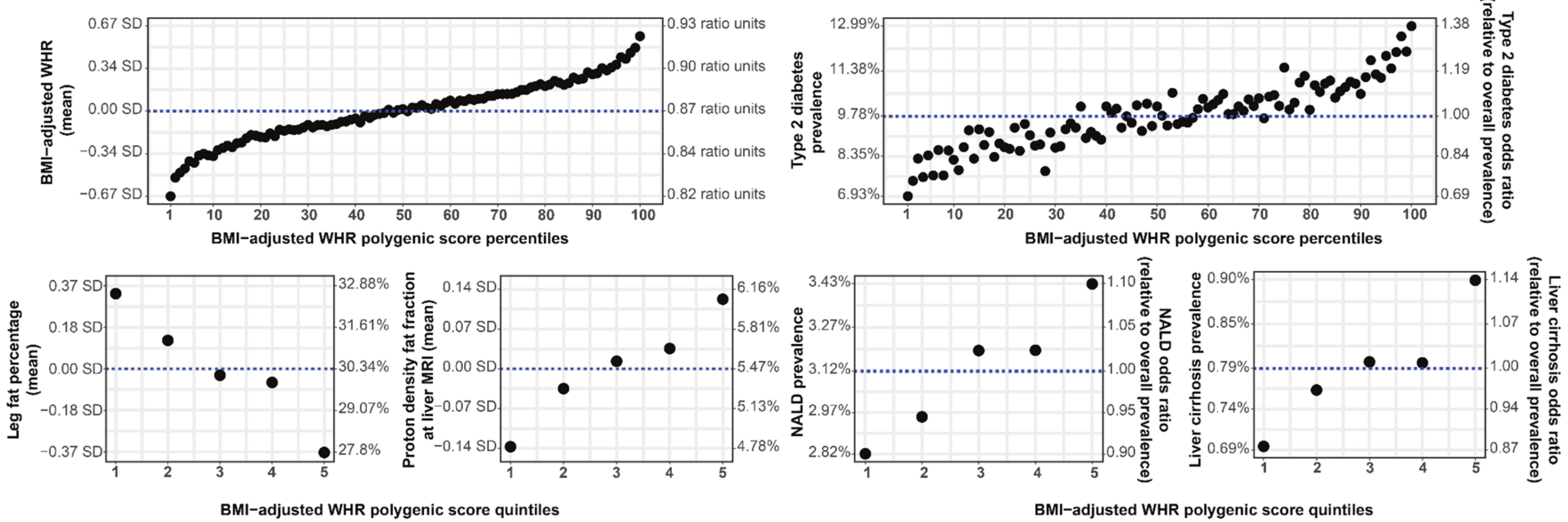

Supplementary Figure 18. Association of a polygenic score for higher BMI-adjusted WHR with fat distribution and type 2 diabetes risk.

Panel A shows associations with BMI-adjusted WHR for quintiles of the BMI-adjusted WHR polygenic score (n=391,810) with the middle quintile used as reference category. Panel B shows associations with type 2 diabetes (38,802 cases and 410,903 controls). Markers represent the beta coefficient (Panel A) or the odds ratio (Panel B) and the error bars represent their 95% confidence intervals. Abbreviations: UKB, UK Biobank; CI, confidence interval; BMI, body mass index; WHR, waist-hip ratio.

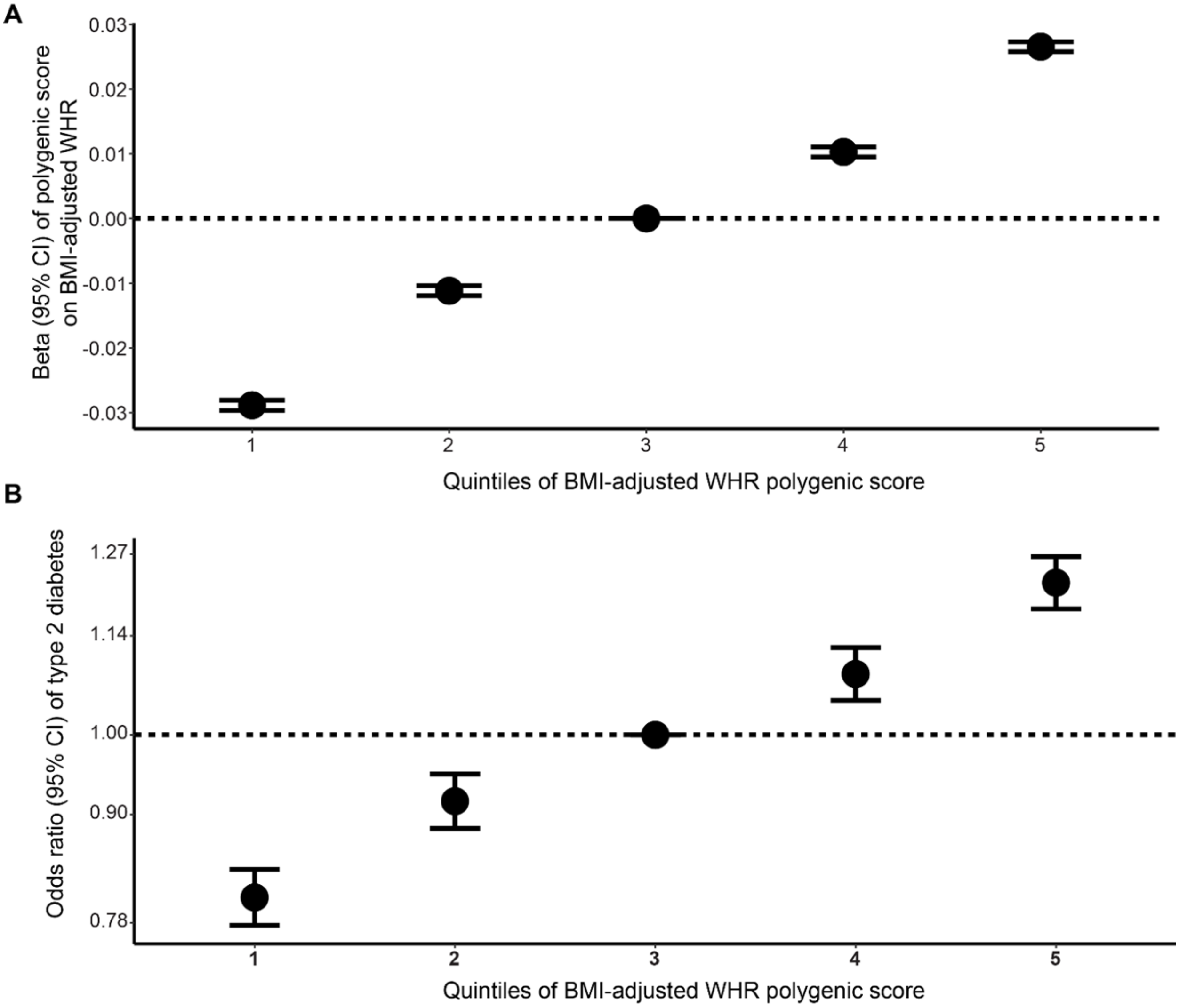

Supplementary Figure 19. Association of a polygenic score for higher BMI-adjusted WHR with refined measures of fat distribution and adipose expandability at DXA.

The figure shows the mean BMI-adjusted visceral-abdominal to gluteofemoral (VA/G) fat mass ratio (left) and mean gluteofemoral fat mass (right) across quintiles of the BMI-adjusted WHR polygenic score. Analyses were performed in the UKB study. Abbreviations: BMI, body mass index; WHR, waist-hip ratio; DXA, Dual-energy X-ray absorptiometry; VA/G, visceral-abdominal to gluteofemoral fat mass ratio; SD, standard deviation; g, gram; UKB, UK Biobank.

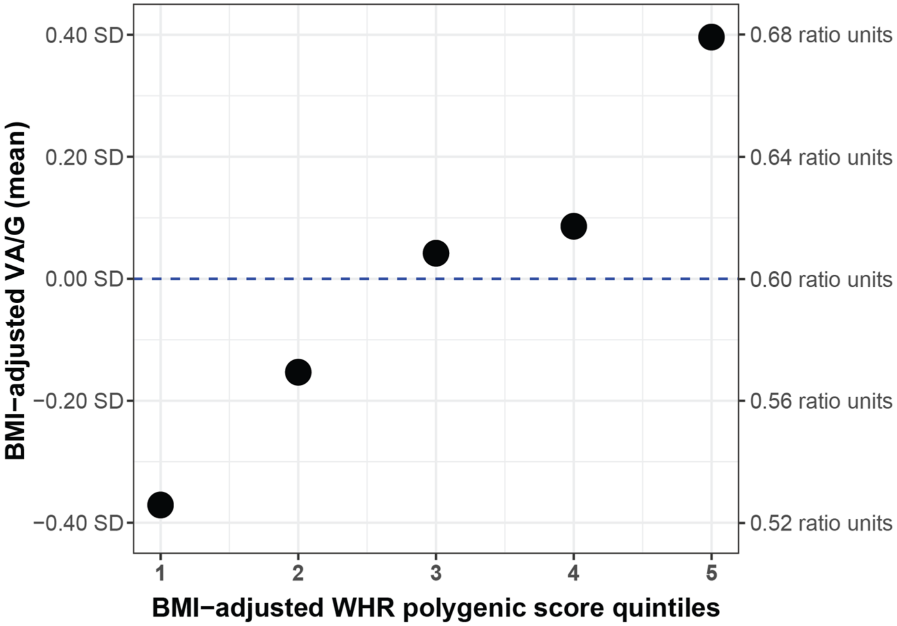

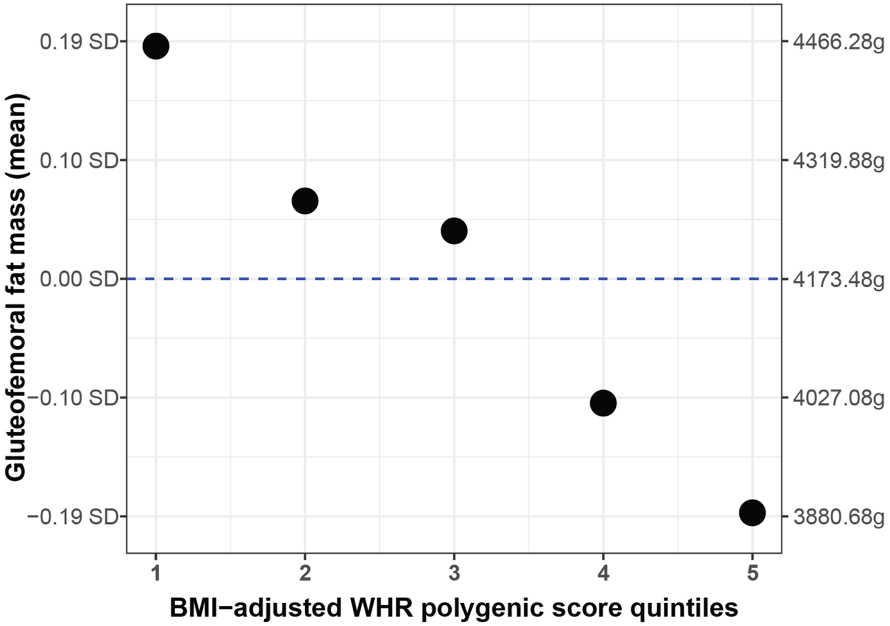

Supplementary Figure 20. Model selection for BMI-adjusted WHR polygenic score in MDCS using GWAS data from a previously published study as the training dataset.

The plot reports the variance explained in the BMI-adjusted WHR phenotype (top panel) and the number of variants included in the score (bottom panel) for each of the different polygenic score generation approaches using the external and independent Shungin et al dataset *(22)* as the training dataset. Analyses were performed in the Malmö Diet and Cancer Study cohort. Abbreviations: GWAS, genome-wide association study; MDCS, Malmö Diet and Cancer Study; BMI-adjusted WHR, waist-hip ratio adjusted for body mass index; Cojo, conditional and joint analysis approach; P+T, clumping and thresholding approach; p, P-value threshold used; pi, assumed proportion of non-zero effect variants; R_sqrd, squared Pearson correlation coefficient threshold used to filter variants; R^2, proportion of variance explained in BMI-adjusted WHR by its polygenic score after covariate adjustment (see **Methods**); log, logarithm

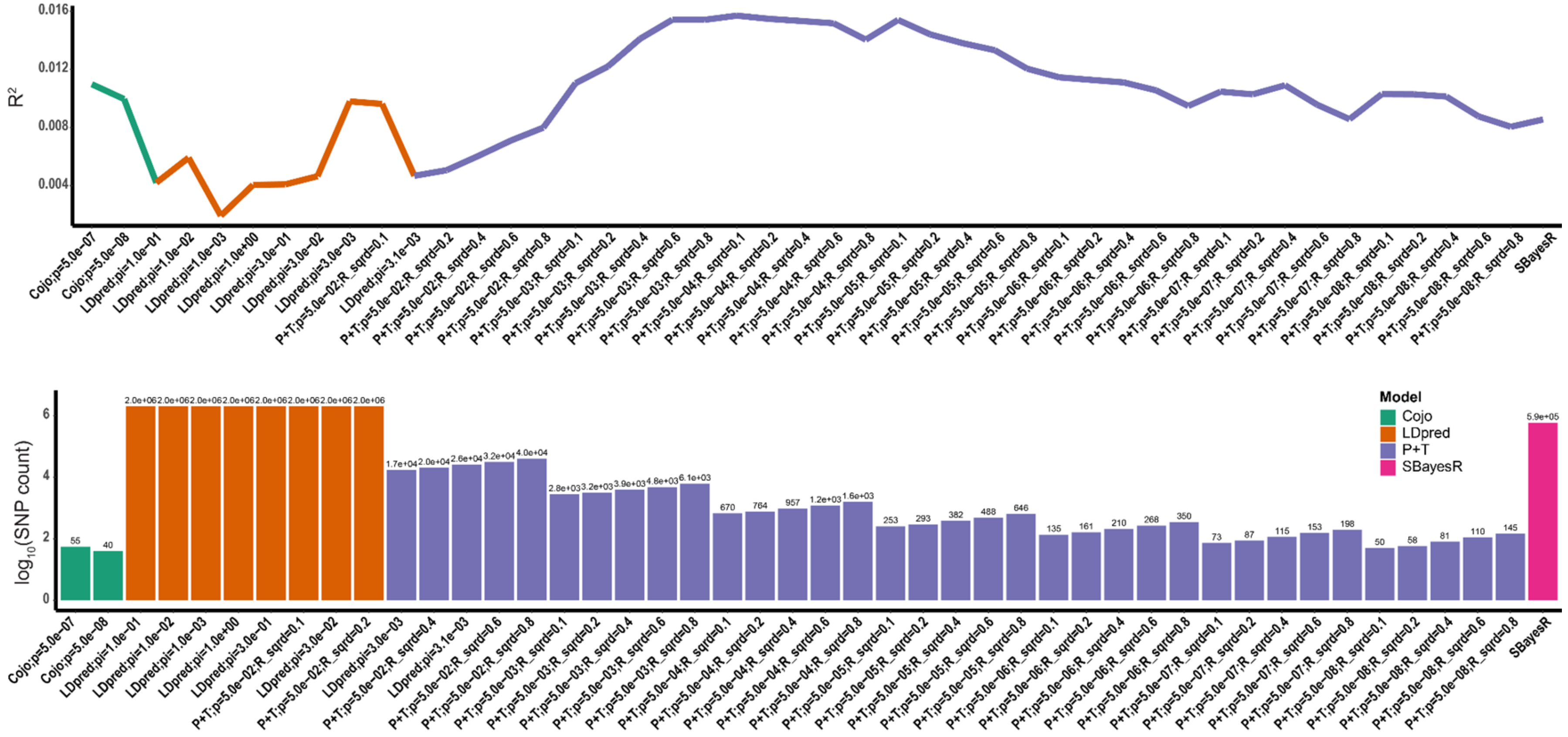

Supplementary Figure 21. Rare predicted loss-of-function variants in *PLIN1* identified by exome sequencing and their distribution on the gene.

A linear model of the *PLIN1* gene with perilipin domain in brown, and an exon track showing the eight exons (in alternating blue and purple) of the gene. Predicted loss of function (pLOF) variants identified by exome-sequencing and included in the gene-burden analysis are shown with number in parenthesis indicating the number of carriers for each allele.

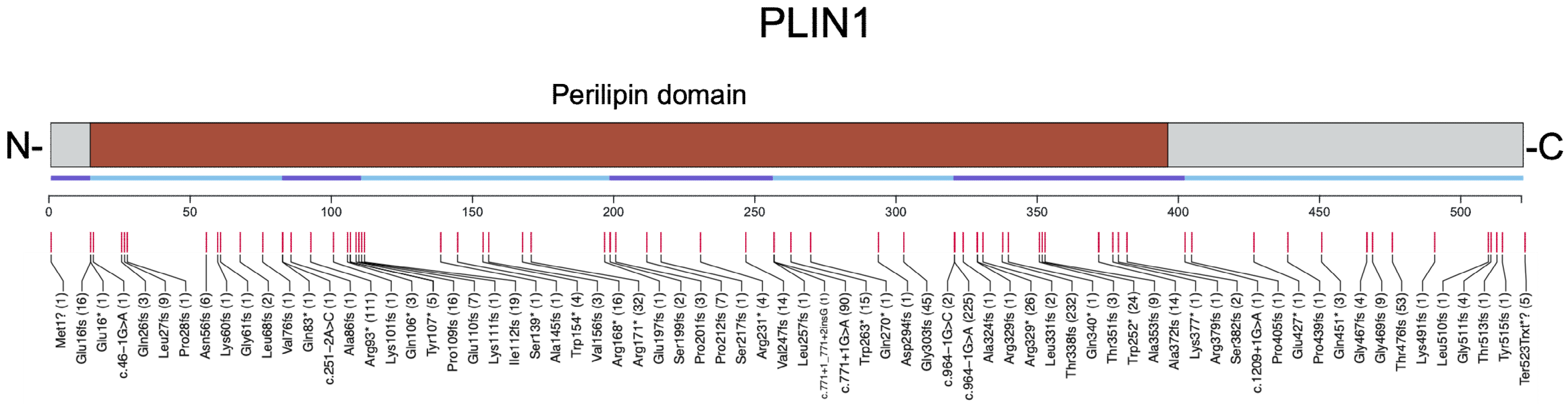

SUPPLEMENTARY REFERENCES

1. S. Gandotra *et al.*, Perilipin deficiency and autosomal dominant partial lipodystrophy. *N Engl J Med* **364**, 740-748 (2011).
2. S. Gandotra *et al.*, Human frame shift mutations affecting the carboxyl terminus of perilipin increase lipolysis by failing to sequester the adipose triglyceride lipase (ATGL) coactivator AB-hydrolase-containing 5 (ABHD5). *J Biol Chem* **286**, 34998-35006 (2011).
3. K. J. Karczewski *et al.*, The mutational constraint spectrum quantified from variation in 141,456 humans. *Nature* **581**, 434-443 (2020).
4. P. J. Thul *et al.*, A subcellular map of the human proteome. *Science* **356**, (2017).
5. D. T. Investigators *et al.*, Effect of rosiglitazone on the frequency of diabetes in patients with impaired glucose tolerance or impaired fasting glucose: a randomised controlled trial. *Lancet* **368**, 1096-1105 (2006).
6. S. E. Kahn *et al.*, Glycemic durability of rosiglitazone, metformin, or glyburide monotherapy. *N Engl J Med* **355**, 2427-2443 (2006).
7. P. Akbari *et al.*, Sequencing of 640,000 exomes identifies GPR75 variants associated with protection from obesity. *Science* **373**, (2021).
8. A. V. Khera *et al.*, Genome-wide polygenic scores for common diseases identify individuals with risk equivalent to monogenic mutations. *Nat Genet* **50**, 1219-1224 (2018).
9. A. V. Khera *et al.*, Polygenic Prediction of Weight and Obesity Trajectories from Birth to Adulthood. *Cell* **177**, 587-596 e589 (2019).
10. D. Shungin *et al.*, New genetic loci link adipose and insulin biology to body fat distribution. *Nature* **518**, 187-196 (2015).
11. Genotype Tissue Expression (GTEx) Project, *Tissue Sampling Sites* (https://www.gtexportal.org/home/samplingSitePage).
