# Supplementary material for: Multiancestry exome sequencing reveals INHBE mutations associated with favorable fat distribution and protection from diabetes
Source: Nat Commun. 2022 Aug 23;13:4844. doi: 10.1038/s41467-022-32398-7 (PMC9399235; doi:10.1038/s41467-022-32398-7)
Supplement: Supplementary file 2 — Description of Additional Supplementary Files [file 41467_2022_32398_MOESM2_ESM.pdf]

Description of Additional Supplementary Files

File Name: Supplementary Data 1
Description: Baseline characteristics of individuals included in exome-wide association study.

File Name: Supplementary Data 2
Description: Sentinel variants identified by fine-mapping in the GWAS analysis of BMI-adjusted WHR.

File Name: Supplementary Data 3
Description: Associations by ancestry and cohort for the genes identified in the gene-burden discovery analysis.

File Name: Supplementary Data 4
Description: Rare coding variants in genes identified in the gene-burden discovery analyses.

File Name: Supplementary Data 5
Description: Associations with visceral to gluteofemoral fat ratio as measured by magnetic resonance imaging (MRI) for the genes identified in the BMI-adjusted WHR discovery analysis.

File Name: Supplementary Data 6
Description: Previously reported associations of rare coding variants with BMI-adjusted WHR.

File Name: Supplementary Data 7
Description: Familial partial lipodystrophy genes and their association with fat distribution in the exome-wide analysis.

File Name: Supplementary Data 8
Description: Associations with waist and hip circumference for the genes identified in the gene-burden discovery analysis.

File Name: Supplementary Data 9
Description: Associations with BMI-adjusted WHR by sex for the genes identified in the gene-burden discovery analyses.

File Name: Supplementary Data 10
Description: Associations with BMI-adjusted WHR in the exome-wide analysis of rare nonsynonymous single variants.

File Name: Supplementary Data 11
Description: Association of INHBE rare pLOF variants with cardiometabolic traits.

File Name: Supplementary Data 12
Description: Association of rare pLOF variants in INHBE with MRI-measured visceral and gluteofemoral fat volume.

File Name: Supplementary Data 13
Description: Association of pLOF variants in INHBE with birthweight and self-reported comparative body size at age 10.

File Name: Supplementary Data 14
Description: Rare pLOF variants in INHBE identified by exome sequencing.

File Name: Supplementary Data 15
Description: Associations of rare nonsynonymous variants in INHBE with BMI-adjusted WHR, after excluding carriers of the SLC26A10-Ser544Asn missense variant.

File Name: Supplementary Data 16
Description: Association of rare nonsynonymous variants in SLC26A10 with BMI-adjusted WHR, after excluding the SLC26A10-Ser544Asn missense variant.

File Name: Supplementary Data 17
Description: Genes showing large-effect associations with favorable fat distribution and their association with type 2 diabetes.

File Name: Supplementary Data 18
Description: Association with type 2 diabetes within obesity- and sex-categories for rare pLOF variants in INHBE.

File Name: Supplementary Data 19
Description: Associations of pLOF variants in INHBE with liver traits and disease.

File Name: Supplementary Data 20
Description: Association of lower favorable fat distribution polygenic scores with type 2 diabetes and non-alcoholic liver disease, adjusted for disease risk factors.

File Name: Supplementary Data 21
Description: Association of rare coding variants with liver phenotypes, type 2 diabetes and coronary artery disease, stratified by sex.

File Name: Supplementary Data 22
Description: Association of rare coding variants and favorable fat distribution polygenic scores with type 2 diabetes, stratified by obesity status.

File Name: Supplementary Data 23
Description: Association results for PPARG mutations.

File Name: Supplementary Data 24
Description: Fat distribution and prevalence of type 2 diabetes in individuals found in high-impact genetic exposure groups including PLIN4 and ANKRD12.

File Name: Supplementary Data 25
Description: Fat distribution and prevalence of type 2 diabetes in individuals found in high-impact genetic exposure groups using polygenic scores generated in a sensitivity analysis.

File Name: Supplementary Data 26
Description: Definitions used for liver disease outcomes.

File Name: Supplementary Data 27
Description: Association of rare pLOF variants in PLIN1 with fat distribution traits.

File Name: Supplementary Data 28

Description: Associations of rare pLOF variants in INHBE or rare pLOF plus deleterious missense variants in PPARG with fracture risk and estimated bone mineral density.
