# Supplementary material for: Multiancestry exome sequencing reveals INHBE mutations associated with favorable fat distribution and protection from diabetes
Source: Nat Commun. 2022 Aug 23;13:4844. doi: 10.1038/s41467-022-32398-7 (PMC9399235; doi:10.1038/s41467-022-32398-7)
Supplement: Supplementary file 5 — Reporting Summary [file 41467_2022_32398_MOESM5_ESM.pdf]

nature portfolio

Corresponding author(s): Luca Lotta

Last updated by author(s): Jul 20, 2022

Reporting Summary

Nature Portfolio wishes to improve the reproducibility of the work that we publish. This form provides structure for consistency and transparency in reporting. For further information on Nature Portfolio policies, see our Editorial Policies and the Editorial Policy Checklist.

Statistics

For all statistical analyses, confirm that the following items are present in the figure legend, table legend, main text, or Methods section.

n/a	Confirmed	
☐	☒	The exact sample size (*n*) for each experimental group/condition, given as a discrete number and unit of measurement
☐	☒	A statement on whether measurements were taken from distinct samples or whether the same sample was measured repeatedly
☐	☒	The statistical test(s) used AND whether they are one- or two-sided<br>*Only common tests should be described solely by name; describe more complex techniques in the Methods section.*
☐	☒	A description of all covariates tested
☐	☒	A description of any assumptions or corrections, such as tests of normality and adjustment for multiple comparisons
☐	☒	A full description of the statistical parameters including central tendency (e.g. means) or other basic estimates (e.g. regression coefficient) AND variation (e.g. standard deviation) or associated estimates of uncertainty (e.g. confidence intervals)
☐	☒	For null hypothesis testing, the test statistic (e.g. *F*, *t*, *r*) with confidence intervals, effect sizes, degrees of freedom and *P* value noted<br>*Give P values as exact values whenever suitable.*
☒	☐	For Bayesian analysis, information on the choice of priors and Markov chain Monte Carlo settings
☒	☐	For hierarchical and complex designs, identification of the appropriate level for tests and full reporting of outcomes
☐	☒	Estimates of effect sizes (e.g. Cohen's *d*, Pearson's *r*), indicating how they were calculated

Our web collection on statistics for biologists contains articles on many of the points above.

Software and code

Policy information about availability of computer code

Data collection	No software was used for data collection.
Data analysis	The association analyses were performed using REGENIE v2 (available at https://github.com/rgcgithub/regenie).<br>Fine-mapping of GWAS data was performed using FINEMAP v1.4.<br>Polygenic scores were derived using GCTA v1.93, LDpred v1.0.11, and sBayesR v2.02.<br>Mendelian randomization analyses were performed using TwoSampleMR v0.5.6 and MendelianRandomization v0.5.1.<br>Liver gene expression data were analysed using edgeR v3.32.1, DESeq2, and apeglm v1.12.0.

For manuscripts utilizing custom algorithms or software that are central to the research but not yet described in published literature, software must be made available to editors and reviewers. We strongly encourage code deposition in a community repository (e.g. GitHub). See the Nature Portfolio guidelines for submitting code & software for further information.

Data

Policy information about availability of data

All manuscripts must include a data availability statement. This statement should provide the following information, where applicable:

- Accession codes, unique identifiers, or web links for publicly available datasets
- A description of any restrictions on data availability
- For clinical datasets or third party data, please ensure that the statement adheres to our policy

The data supporting the findings of this manuscript are reported in the main text, in the figures, in the supplementary materials, and are tabulated in Table 1, Table 2 and Supplementary Data 1 to 28. UKB individual-level genotypic and phenotypic data may be accessed by approved investigators via the UK Biobank study (www.ukbiobank.ac.uk/). Additional information about registration for access to the data are available at www.ukbiobank.ac.uk/register-apply/. Data access for

approved applications requires a data transfer agreement between the researcher's institution and UK Biobank, the terms of which are available on the UK Biobank website (www.ukbiobank.ac.uk/media/ezrderzw/applicant-mta.pdf). MCPS data may be available to qualified non-commercial researchers to reproduce results reported in this manuscript by emailing mcps-access@ndph.ox.ac.uk. The data access policy can be downloaded from https://www.ctsu.ox.ac.uk/research/prospective-blood-based-study-of-150-000-individuals-in-mexico. MDCS data may be available to qualified academic non-commercial researchers to reproduce results reported in this manuscript through the portal at https://www.malmo-kohorter.lu.se/malmo-cohorts, following the principles outlined in this policy https://www.malmo-kohorter.lu.se/sites/malmo-kohorter.lu.se/files/mdcs_mpp_mos_request_form_vermar20.doc. eQTL summary statistics may be downloaded from the GTEx portal (https://gtexportal.org/). The GRCh38 reference assembly may be accessed from the Genome Reference Consortium (https://www.ncbi.nlm.nih.gov/grc).

Field-specific reporting

Please select the one below that is the best fit for your research. If you are not sure, read the appropriate sections before making your selection.

☒ Life sciences ☐ Behavioural & social sciences ☐ Ecological, evolutionary & environmental sciences

For a reference copy of the document with all sections, see nature.com/documents/nr-reporting-summary-flat.pdf

Life sciences study design

All studies must disclose on these points even when the disclosure is negative.

Sample size

The sample size was not predetermined, and the largest available sample size was included where possible.

Data exclusions

All available samples that passed genotype and phenotype QC were included in association analyses. Phenotype selection and QC was performed as described in methods section "Phenotype definitions". Variant level QC was performed as described in methods section "Whole exome sequencing and genotyping data".

Replication

Not applicable.

Randomization

This is an observational study, hence randomization was not applicable.

Blinding

This is an observational study, hence blinding was not applicable.

Reporting for specific materials, systems and methods

We require information from authors about some types of materials, experimental systems and methods used in many studies. Here, indicate whether each material, system or method listed is relevant to your study. If you are not sure if a list item applies to your research, read the appropriate section before selecting a response.

Materials & experimental systems

n/a	Involved in the study	
☐	☒	Antibodies
☐	☒	Eukaryotic cell lines
☒	☐	Palaeontology and archaeology
☒	☐	Animals and other organisms
☐	☒	Human research participants
☒	☐	Clinical data
☒	☐	Dual use research of concern

Methods

n/a	Involved in the study	
☒	☐	ChIP-seq
☒	☐	Flow cytometry
☒	☐	MRI-based neuroimaging

Antibodies

Antibodies used

INHBE: Novus Biologicals, H00083729-B01P
Anti-mouse secondary antibody: Cell Signaling 7076

Validation

The INHBE antibody was experimentally validated for Western Blot detection of human INHBE via detection of GST-tagged full length human INHBE recombinant protein (Abnova, H00083729-P01; data shown in the figures) and commercially available human INHBE-ORF expression vector (Sino Biologicals, HG10645-UT) transfected expiCHO cell lysate (data not shown) during Western Blotting. Additional information supplied by the manufacturer: "Antibody reactive against Recombinant Protein with GST tag on ELISA and Western Blot and also on transfected lysate in western blot"; image of "Western Blot: INHBE Antibody [H00083729-B01P] - Analysis of INHBE expression in transfected 293T cell line by INHBE polyclonal antibody" showing detection in lysate from 293T cells transfected with INHBE but not in non-transfected 293T cell lysate.

Eukaryotic cell lines

Policy information about cell lines

Cell line source(s)	ExpiCHO-S: Thermo Fisher (https://www.thermofisher.com/order/catalog/product/A29127).
Authentication	The ExpiCHO-S cell line is derived from a non-engineered sub-clone that was screened and isolated from CHO-S Chinese hamster ovary (CHO) cells. This line was obtained from the manufacturer and was not otherwise authenticated.
Mycoplasma contamination	The ExpiCHO-S cells were not tested for mycoplasma contamination.
Commonly misidentified lines (See ICLAC register)	ExpiCHO-S cell lines are not listed on ICLAC as misidentified.

Human research participants

Policy information about studies involving human research participants

Population characteristics	Exome-wide analyses were performed in UK Biobank (UKB), Malmö Diet and Cancer study (MDCS), and Mexico City Prospective Study (MCPS). UKB is a population-based cohort of people 40-69 years of age recruited in the UK between 2006-2010. A total of 429,442 European, 10,115 South Asian, 8,948 African, 2,203 East Asian, 604 American ancestry participants with exome sequencing and phenotypic data were included. MDCS is a population-based cohort of 44 to 73 years-old people living in Malmö (Sweden) and recruited in 1991–1996. A total of 28,875 European ancestry participants were included. MCPS is a population-based cohort of people aged 35 years or older, recruited from two urban districts in Mexico City in 1998-2004. A total of 138,188 participants of Admixed American ancestry were included. Ancillary analyses included association results from 109,909 participants in the Geisinger Health System MyCode and DiscovEHR collaborations (GHS), 28,338 participants in the Mount Sinai BioMe biobank cohort (BioMe; mean age, 55 years; 59% women), and 15,046 participants in the University of Pennsylvania PennMedicine Biobank cohort (mean age, 63 years; 52% women). General population characteristics for each cohort included in the exome-wide association analysis are shown in Supplementary Data 1.
Recruitment	All studies included in the exome-wide discovery analaysis were population-based (see previous paragraph). Further details on the study design for each cohort can be found in various publications:<br>- UKB: Sudlow et al, PLoS Med 2015 and Bycroft et al, Nature 2018;<br>- MDCS: Berglund et al, J Intern Med 1993;<br>- MCPS: Tapia-Conyer et al, Int J Epidemiol 2006.
Ethics oversight	Ethical approval for the UKB was previously obtained from the North West Centre for Research Ethics Committee (11/NW/0382). The work described herein was approved by UK Biobank under application number 26041. The MCPS study was approved by the Mexican Ministry of Health, the Mexican National Council for Science and Technology, and the University of Oxford. The MDCS study was approved by the Regional Ethics Committee at Lund University. Approval for DiscovEHR analyses was provided by the Geisinger Health System Institutional Review Board under project number 2006-0258. Approval for the University of Pennsylvania Penn Medicine Biobank was provided by the Institutional Review Board of the University of Pennsylvania. Mount Sinai BioMe biobank cohort was approved by the Icahn School of Medicine at Mount Sinai's Institutional Review Board. All participants provided informed consent.

Note that full information on the approval of the study protocol must also be provided in the manuscript.
